# Postural orthostatic tachycardia syndrome is the most frequent cardiovascular autonomic disorder following COVID-19 infection or vaccination

**DOI:** 10.1007/s00415-025-13518-x

**Published:** 2025-11-22

**Authors:** Fabian Leys, Mara Verginer, Elias Kirchler, Loraine Marino, Georg Goebel, Nicole Campese, Sabine Eschlböck, Susanne Duerr, Gregor Broessner, Atbin Djamshidian-Tehrani, Anna Heidbreder, Birgit Högl, Maria-Sophie Rothmund-Grenier, Katharina Hüfner, Sarah Iglseder, Wolfgang Löscher, Ambra Stefani, Julia Wanschitz, Günter Weiss, Laura Zamarian, Judith Löffler-Ragg, Raimund Helbok, Stefan Kiechl, Roberta Granata, Gregor K. Wenning, Alessandra Fanciulli

**Affiliations:** 1 Department of Neurology, Medical University of Innsbruck, Innsbruck, Austria; 2 Institute of Medical Statistics and Informatics, Medical University of Innsbruck, Innsbruck, Austria; 3 Department of Neurology, Hochzirl-Natters Hospital, Zirl, Austria; 4 Department of Neurology, Kepler University Clinic, Linz, Austria; 5 Department of Psychiatry, Psychotherapy, Psychosomatics and Medical Psychology, University Hospital of Psychiatry II, Medical University of Innsbruck, Innsbruck, Austria; 6 Department of Internal Medicine II, Medical University of Innsbruck, Innsbruck, Austria; 7 VASCage - Centre On Clinical Stroke Research, Innsbruck, Austria

**Keywords:** POTS, Cardiovascular autonomic disorders, COVID-19, SARS-CoV-2, Infection, Vaccination

## Abstract

**Background:**

Cardiovascular autonomic disorders (CAD) were described following COVID-19 infection and vaccination, but previous reports were limited in size and follow-up. Here, we aimed to investigate the type and frequency of newly diagnosed and exacerbated CAD following COVID-19 infection or vaccination, and assessed their associated autonomic and non-autonomic complaints, applied treatment, and clinical outcome at last follow-up.

**Methods:**

Medical records of individuals referred to the Innsbruck Dysautonomia Center between March 2020 and March 2023 were reviewed for new onset of orthostatic intolerance, recurrent syncope, *OR* exacerbation of previously diagnosed CAD within 6 weeks from a passed COVID-19 infection or vaccination.

**Results:**

Following COVID-19 infection (*n* = 75), 22 (29%) individuals were diagnosed with postural orthostatic tachycardia syndrome (POTS), 12 (16%) with vasovagal syncope (VVS), 1 with delayed and 1 with transient orthostatic hypotension (OH). Following COVID-19 vaccination (*n* = 26), 11 (42%) POTS, 2 (8%) VVS, and 3 (12%) transient OH cases were newly diagnosed. In half of newly referred individuals (*n* = 49/101, 49%), the diagnostic workup excluded any CAD. VVS was the most frequently exacerbated CAD (*n* = 8/19, 42%). Non-pharmacological measures were recommended to all newly diagnosed CAD, with one-third additionally receiving pharmacotherapy. Follow-up was available in 42 (81%) individuals with newly diagnosed CAD, with a symptomatic improvement observed in 26 (62%) cases.

**Conclusion:**

A specialized diagnostic workup is pivotal to diagnose or exclude CAD in individuals with new-onset orthostatic intolerance or recurrent syncope following COVID-19 infection or vaccination. A multimodal treatment approach can achieve a symptomatic improvement in a substantial proportion of affected individuals.

**Supplementary Information:**

The online version contains supplementary material available at 10.1007/s00415-025-13518-x.

## Introduction

Following its outbreak in late 2019, the SARS-CoV-2 virus and the subsequent COVID-19 pandemic placed major strain on healthcare systems, medical research, economies, and social life worldwide [1–3]. The clinical spectrum of COVID-19 is broad and can vary from asymptomatic to critical illness [4]. Individuals with COVID-19 typically present with a respiratory syndrome, fever, and headache, but may also experience neurological manifestations [5–7], including symptoms reminiscent of autonomic nervous system (ANS) dysfunction [8, 9].

When intact, the ANS and its multiple components manage and maintain the human body’s homeostasis by controlling the heart and blood vessels, respiration, digestion, body temperature, and sweating, as well as the bladder and reproductive organs [10]. Autonomic dysfunction, or dysautonomia, refers to a condition associated with malfunctioning of one or more components of the ANS [10] and can occur at different stages of COVID-19 [11].

An acute SARS-CoV-2 infection and its concomitant immune response may immediately affect the ANS [12], with dehydration and fever possibly leading to, or aggravating, pre-existing orthostatic hypotension (OH), thereby increasing the risk of orthostatic syncope and falls [13]. Likewise, lower blood pressure (BP) levels due to fluid loss may exacerbate postural tachycardia and increase the risk of vasovagal syncope (VVS), while intense coughing may directly trigger reflex syncope [13]. On the other hand, studies have shown that COVID-19 can lead to new-onset autonomic dysfunction or exacerbate pre-existing ANS disorders [11, 14–17].

The underlying mechanisms remain unknown [18, 19], but viral and/or immune-mediated factors, as well as prolonged bed rest and physical deconditioning, may all play a substantial role in the development or exacerbation of cardiovascular autonomic disorders (CAD), such as postural orthostatic tachycardia syndrome (POTS), OH, and VVS [1, 11, 15, 16].

Epidemiological data on CAD generally is scarce and prevalence varies depending on age. In the general population, estimated pre-pandemic prevalences of POTS, OH, and VVS account for 0.2–1% [20], 8–32% [21], and 16% [22], respectively. Early studies that have investigated autonomic dysfunction in people following COVID-19 infection reported CAD as frequent sequalae; however, these were mainly single case reports or comprised small series with poor diagnostic accuracy, lacking structured clinical (autonomic) assessments and comprehensive follow-up [8, 12, 14, 15, 23–26]. While CAD have been in the spotlight so far [2, 8, 26], non-cardiovascular autonomic disturbances may also develop following COVID-19 infection [14, 27]; and vaccination against COVID-19 could likewise cause or exacerbate ANS dysfunction, but even a less number of studies have addressed the matter [28–33].

Here, we aimed to investigate the type and frequency of newly diagnosed and exacerbated CAD following COVID-19 infection or vaccination in a large case series referred to the Innsbruck Dysautonomia Center. In the identified cases, we additionally sought to study the frequency of associated autonomic and non-autonomic complaints, applied treatment, and assessed the clinical outcome at the last available follow-up.

## Methods

### Study population and data acquisition

Medical records of individuals referred to the Innsbruck Dysautonomia Center between March 2020 and March 2023 were reviewed for new onset of orthostatic intolerance (OI), recurrent syncope, *OR* exacerbation of previously diagnosed CAD within 6 weeks from a passed COVID-19 infection [34] *OR* COVID-19 vaccination. Individuals were typically referred in the context of routine clinical care by practicing physicians or through a newly established post-COVID regional network (i.e., “*post-COVID Tyrol program”* coordinated by the Regional State Institute for Integrated Care, https://www.postcovid.tirol/). Individuals with previously diagnosed CAD were seen within scheduled or on-demand follow-up visits.

Cases lacking documentation (i.e., insufficient to establish a temporal relationship between the orthostatic symptom onset *OR* worsening of known CAD and the passed COVID-19 infection *OR* vaccination) or cardiovascular autonomic function test (CAFT) data were excluded. CAFT, 24-h ambulatory BP monitoring (24-h ABPM), and quantitative sudomotor axon reflex test (QSART) were performed at the Innsbruck Dysautonomia Center under standardized conditions according to protocols published elsewhere [35–37]. The CAFT battery comprised a head-up tilt and active standing test, as well as the Valsalva maneuver and deep metronomic breathing.

Clinical data of the identified cases were subsequently collected from available electronic and handwritten medical records and included:

- Cardiovascular autonomic diagnosis (i.e., POTS [20], VVS [38], classic, delayed, or transient OH [38–40], counterchecked against current diagnostic criteria, whereas individuals with new-onset OI but unremarkable CAFT findings were classified as OI without CAD [i.e., OIw/oCAD]);
- Clinical–demographic information (i.e., age, sex, height, weight, and BMI);
- Information regarding COVID-19 infection (i.e., infection severity according to the WHO classification [34], latency to orthostatic symptom onset/exacerbation, vaccination status at the time of infection);
- Information regarding COVID-19 vaccination (i.e., number and last type of Vaccine received before orthostatic symptom onset/exacerbation, latency to orthostatic symptom onset/exacerbation, total number and types of vaccines received);
- Additional autonomic complaints (i.e., accompanying thermoregulatory/sudomotor, respiratory, gastrointestinal, vasomotor, and/or urogenital complaints);
- Additional non-autonomic complaints and abnormalities (i.e., fatigue, neurocognitive, psychiatric, headache, sleep, cardiac, pulmonary, olfactory, neuromuscular, gustatory, and/or laboratory complaints and abnormalities);
- Pre-existing comorbidities;
- Applied treatment for cardiovascular autonomic complaints (i.e., in newly diagnosed CAD, start of any behavioral, non-pharmacological and pharmacological measures; in exacerbated CAD, add-on, or increase of non-pharmacological and pharmacological measures);
- Additional autonomic function assessment data (i.e., results of 24-h ABPM and QSART; BP behavior during the Valsalva maneuver; sudomotor and cardiovagal composite autonomic severity scores [CASS] [35]; as well as co-occurrence of transient OH in POTS, VVS, and classic and delayed OH cases [38–40]);
- Follow-up information (i.e., follow-up time, symptomatic course [improved vs. not improved], and, in POTS cases, presence of postural orthostatic tachycardia during follow-up active standing test).

### Grading of causal association

Following provisional definitions linking COVID-19 to neurological disease [41], the association between newly diagnosed CAD and COVID-19 infection was labeled as “probable”, if SARS-CoV-2 RNA or antibody evidence of acute SARS-CoV-2 infection was detected, symptoms developed within 6 weeks from the infection, and no other common causes of CAD were found. The causal association was deemed "possible", if symptoms occurred within 6 weeks, but other putative causes were present. The same approach was used to determine the association between newly diagnosed CAD following COVID-19 vaccination.

A detailed description of the data retrieval strategy, definitions, and variables is provided in Supplementary Table 1.

## Statistical analysis

Qualitative variables were summarized by absolute frequency (percentage) and quantitative variables by either mean (± standard deviation) or median [25^th^; 75^th^ percentile]. Absent information was treated as “missing data”; the relative frequencies reported correspond to the proportion of available data. Qualitative variables were analyzed with the Pearson’s Chi-square, Fisher’s exact, or Fisher-Freeman-Halton test, where appropriate. The Shapiro-Wilk test was used to determine the distribution of quantitative variables. Depending on the data distribution, differences in quantitative variables were assessed with the Student’s *t* or Mann-Whitney *U* test. For groupwise comparisons, at least 5 observations per group were required for performing comparative analyses.

First, we studied the type and frequency of cardiovascular autonomic diagnoses (i.e., POTS, VVS, classic, delayed, transient OH; or OIw/oCAD) following COVID-19 infection (“post-COVID”) or vaccination (“post-vaccination”). We then performed within-group analyses among the newly diagnosed CAD and OIw/oCAD (in the post-COVID and post-vaccination cohorts, respectively), focusing on demographics, COVID-19 infection or vaccination-related information, frequency and type of additional autonomic and non-autonomic complaints, comorbidities, applied treatment, and the clinical outcome at last available follow-up. In a subanalysis, post-COVID newly diagnosed CAD (including POTS, VVS, delayed and transient OH cases) were compared depending on their symptomatic outcome at last available follow-up (i.e., improved vs. not-improved). Additionally, we conducted a between-group analysis of post-COVID versus post-vaccination newly diagnosed POTS cases and compared improved versus not-improved cases among them (in both post-COVID and post-vaccination cases combined, and in post-COVID POTS specifically).

Statistical significance was set at a two-tailed p-value of < 0.05, with a blockwise Bonferroni correction applied to multiple testing. The statistical analysis was performed with IBM SPSS® Statistics V29.0 (IBM Corporation, Armonk, NY, USA). Supplementary Fig. 1 summarizes the full analytic approach.

### Data sharing

The first and last authors take responsibility for the integrity of the data presented herewith. The data supporting the findings of this study are available upon reasonable request from any qualified investigator and approval by the responsible bodies.

## Results

We screened 1081 individuals referred to our center between March 2020 and March 2023 and identified 160 individuals who were evaluated/treated in the context of COVID-19 (15%). Thereof, 48 individuals did not fulfill the inclusion criteria (22%) or rather met exclusion criteria (8%), resulting in 112 individuals included in the study (70%). Eight individuals were referred twice at different time points and for different reasons, totaling 120 cases (73% female) with a mean age of 41 (SD ± 14) years.

### Newly diagnosed CAD following COVID-19 infection and vaccination

Following COVID-19 infection, 22 individuals were newly diagnosed with POTS, 12 with VVS, and 1 with delayed and 1 with transient OH, whereas in 39 cases presenting with new-onset OI the diagnostic workup excluded any CAD (i.e., OIw/oCAD). In most newly diagnosed CAD, the causal association with the passed COVID-19 infection was deemed probable (34 out of 36, 94%).

Following COVID-19 vaccination, 11 POTS, 2 VVS, and 3 transient OH cases were newly diagnosed, while 10 cases were classified as OIw/oCAD. Fourteen of the 16 newly diagnosed CAD following COVID-19 vaccination were graded as probably associated with the prior vaccination (88%).

**Fig. 1:**
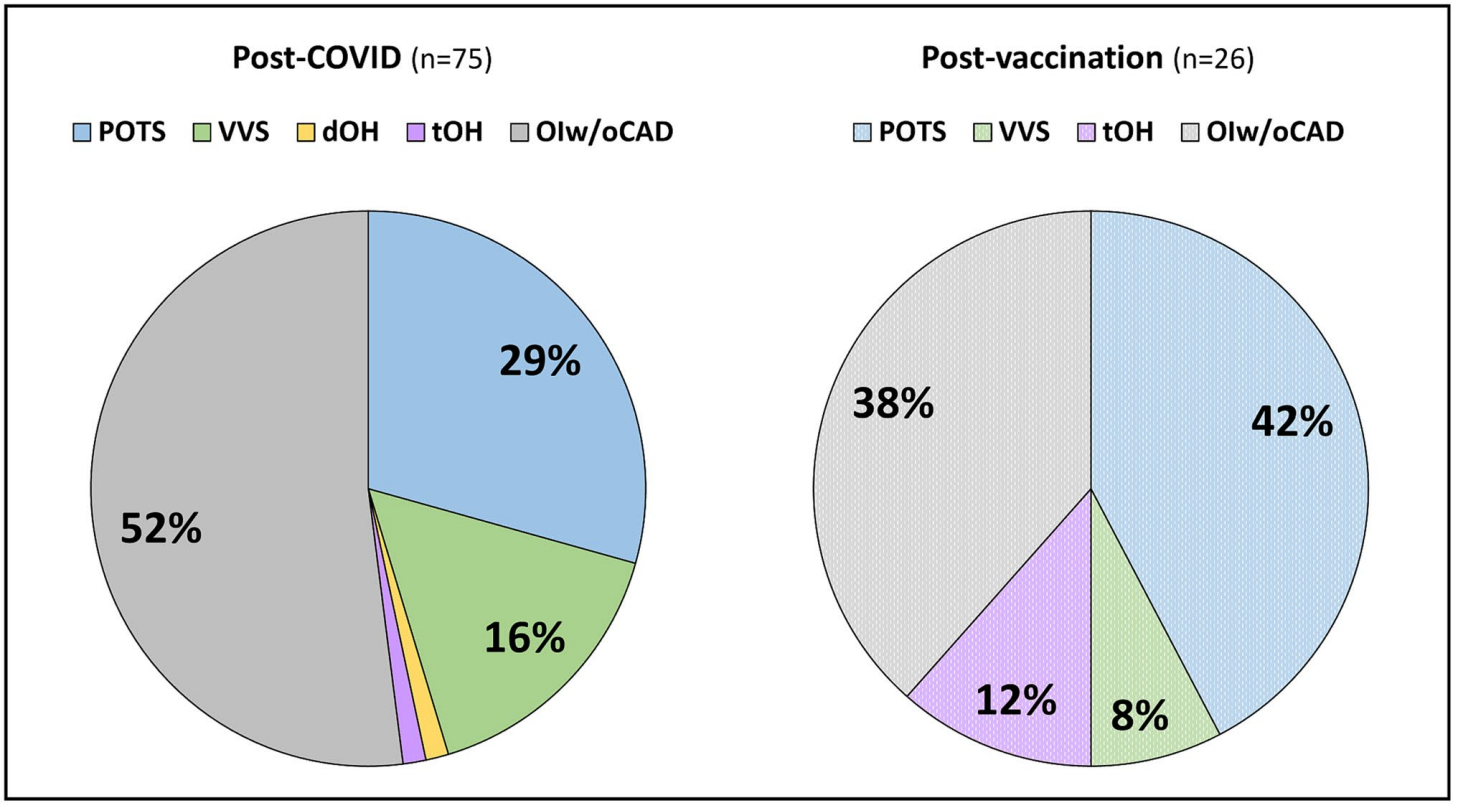
Final diagnosis of individuals referred for suspected CAD following COVID-19 infection of vaccination. Abbreviations: CAD, cardiovascular autonomic disorder; dOH, delayed orthostatic hypotension; OIw/oCAD, orthostatic intolerance without CAD; POTS, postural orthostatic tachycardia syndrome; tOH, transient orthostatic hypotension; VVS, vasovagal syncope. Created with Microsoft Office PowerPoint 2016

Figure 1 illustrates the types and frequencies of newly diagnosed CAD and cases with OIw/oCAD following COVID-19 infection and vaccination.

Among all post-COVID and post-vaccination newly diagnosed CAD, the median latency of symptom onset was 0 (0; 2) weeks. Post-COVID POTS cases were younger than VVS and OIw/oCAD ones (33 ± 8 vs. 45 ± 12 and 43 ± 14 years; *p* = 0.003 and *p* = 0.004, respectively) and, at the time of SARS-CoV-2 infection, post-COVID POTS cases were more frequently vaccinated against COVID-19 than post-COVID VVS (75% vs. 17%; *p* = 0.003).

Post-COVID POTS cases reported a higher number of additional autonomic complaints than post-COVID OIw/oCAD (100% vs. 60%; *p* = 0.001). Likewise, post-vaccination POTS reported more frequently thermoregulatory/sudomotor complaints than post-vaccination OIw/oCAD (100% vs. 25%; *p* = 0.002).

Additional non-autonomic complaints and abnormalities were commonly observed in both the newly diagnosed post-COVID and post-vaccination cohorts. Beyond laboratory abnormalities, the most frequently associated non-autonomic complaints comprised fatigue, neurocognitive disturbances and headache, with cumulative frequencies of 80 (92%), 63 (75%), and 55 (67%), respectively, but no significant differences observed between any newly diagnosed CAD versus OIw/oCAD following COVID-19 infection or vaccination. Post-COVID and post-vaccination associated psychiatric disorders were documented in 30 cases (30%), with depressive (ICD Code: F32 and F33; *n* = 11) and adjustment disorders (ICD Code: F43.2; *n* = 10) representing the most frequent diagnoses, both in post-COVID and post-vaccination newly diagnosed CAD and OIw/oCAD.

Behavioral and non-pharmacological measures to combat OI and recurrent syncope were recommended to all newly diagnosed CAD cases, with approximately one-third additionally receiving pharmacological treatment (*n* = 16, 32%).

Follow-up was available in 42 (81%) of post-COVID and post-vaccination newly diagnosed CAD (median follow-up time 7 [4; 10] months), with a marked orthostatic symptom improvement or full recovery documented in 26 (62%) cases. When comparing cases with versus without symptomatic improvement at last available follow-up, we did not observe any significant differences in the baseline clinical-demographic characteristics (Supplementary Table 2).

Tables 1, 2 provide a comparison of the clinical-demographic characteristics of newly diagnosed CAD and OIw/oCAD cases following COVID-19 infection or vaccination, respectively.

**Table 1:** Newly diagnosed CAD following COVID-19 infection

	Cardiovascular autonomic disorders			*p*		
Newly diagnosed after COVID-19 infection	POTS (N = 22)	VVS (N = 12)	OIw/oCAD (N = 39)	POTS vs. VVS	POTS vs. OIw/oCAD	VVS vs. OIw/oCAD
**Demographics**						
Age—years	33 ± 8	45 ± 12	43 ± 14	0.003*	0.004*	0.737
Female sex—n (%)	19 (86)	10 (83)	25 (64)	1.000	0.079	0.296
Height—cm	166 ± 7	163 ± 8	172 ± 7	0.659	0.010	0.005*
Weight—kg	60 ± 10	69 ± 18	71 ± 19	1.000	0.177	0.756
BMI—kg/m^2^	22 [19; 24]	23 [22; 32]	22 [21; 25]	0.080	0.500	0.496
**COVID-19 infection**						
Infection severity—n (%)				0.388	1.000	0.239
Mild	8 (53)	4 (44)	16 (53)			
Moderate	7 (47)	3 (33)	13 (43)			
Severe	0	1 (11)	1 (3)			
Critical	0	1 (11)	0			
Latency to orthostatic symptom onset—weeks	0 [0; 3]	0 [0; 2]	0 [0; 2]	0.748	0.947	0.889
Vaccinated at the time of infection—n (%)	15 (75)	2 (17)	17 (44)	0.003**	0.029	0.171
COVID-19 vaccinations—total n	3 [2; 3]	3 [2; 3]	3 [2; 3]	1.000	0.820	0.560
**Additional post-COVID autonomic complaints**						
Additional autonomic complaints—n (%)	19 (100)	7 (88)	18 (60)	0.296	0.001***	0.222
Thermoregulatory/sudomotor—n (%)	10 (53)	5 (63)	8 (29)	0.696	0.130	0.107
Respiratory—n (%)	6 (33)	0	4 (13)	0.541	0.145	1.000
Gastrointestinal—n (%)	6 (30)	5 (56)	10 (31)	0.237	0.924	0.248
Vasomotor—n (%)	4 (25)	0	3 (11)	0.538	0.394	1.000
Urogenital—n (%)	2 (11)	2 (29)	1 (4)	0.555	0.562	0.101
**Additional post-COVID non-autonomic complaints and abnormalities**						
Additional non-autonomic complaints/abnormalities—n (%)	22 (100)	12 (100)	38 (100)	-	-	-
Additional non-autonomic complaints/abnormalities—n	5 [3; 7]	6 [3; 9]	4 [3; 7]	0.278	0.790	0.207
Fatigue—n (%)	19 (95)	10 (100)	30 (86)	1.000	0.399	0.571
Neurocognitive—n (%)	16 (76)	8 (80)	27 (84)	1.000	0.492	1.000
Psychiatric—n (%)	5 (23)	6 (50)	10 (26)	0.138	1.000	0.112
Headache—n (%)	13 (72)	7 (78)	21 (66)	1.000	0.757	0.692
Sleep—n (%)	12 (86)	5 (63)	15 (52)	0.309	0.045	0.701
Cardiac—n (%)	4 (25)	3 (30)	4 (14)	1.000	0.434	0.351
Pulmonary—n (%)	7 (41)	6 (86)	10 (39)	0.078	0.859	0.039
Olfactory—n (%)	4 (31)	6 (75)	11 (38)	0.080	0.739	0.109
Neuromuscular—n (%)	4 (25)	1 (17)	4 (14)	1.000	0.434	1.000
Gustatory—n (%)	3 (25)	7 (88)	10 (36)	0.020	0.716	0.016
Other—n (%)	5 (39)	5 (71)	18 (58)	0.350	0.325	0.681
Laboratory—n (%)	16 (80)	10 (91)	20 (65)	0.631	0.348	0.133
**Comorbidities**						
Comorbidities—n (%)	17 (77)	11 (92)	29 (74)	0.389	1.000	0.422
Comorbidities—n	2 [1; 3]	3 [1; 5]	1 [0; 3]	0.882	0.753	0.482
**Treatment**						
Behavioral measures—n (%)	22 (100)	10 (100)	NA	–	–	–
Non-pharmacological measures—n (%)	22 (100)	10 (100)	NA	–	–	–
Pharmacological measures—n (%)	6 (27)	2 (20)	NA	1.000	–	–
**Follow-up**						
Available—n (%)	19 (86)	9 (75)	24 (62)	0.641	0.047	0.502
Follow-up time—months	7 [4; 10]	10 [5; 14]	5 [2; 9]	0.140	0.172	0.269
Improved symptomatic course—n (%)	13 (68)	5 (56)	12 (52)	0.677	0.286	1.000

Quantitative values are shown as mean ± SD or median [25^th^, 75^th^ percentile]; qualitative variables as n (%). Distribution was assessed with the Shapiro-Wilk test. Bold font indicates significant p-values after blockwise Bonferroni correction (* p<0.010; ** p<0.013; *** p<0.008).

Data of delayed (n = 1, male, 64 years old) and transient (n = 1, female, 37 years old) OH cases are not shown in the table above due to the low sample size preventing any meaningful statistical description. Both were vaccinated at the time of COVID-19 infection, did not receive pharmacological treatment, and did not show a symptomatic improvement at the last available follow-up. Likewise, due to limited data availability, additional autonomic function assessment data is shown for newly diagnosed POTS only (see Table 3) Abbreviations: CAD, cardiovascular autonomic disorder; COVID-19, coronavirus disease 2019; N/n, number; OH, orthostatic hypotension; OIw/oCAD, orthostatic intolerance without CAD; POTS, postural orthostatic tachycardia syndrome; VVS, vasovagal syncope.

**Table 2:** Newly diagnosed CAD following COVID-19 vaccination

Cardiovascular autonomic disorders			*p*
Newly diagnosed after COVID-19 vaccination	POTS (N = 11)	OIw/oCAD (N = 10)	POTS vs OIw/oCAD
**Demographics**			
Age—years	32 ± 10	47 ± 14	0.011
Female sex—n (%)	8 (73)	8 (80)	1.000
Height—cm	169 ± 4	168 ± 6	0.678
Weight—kg	55 ± 7	63 ± 10	0.136
BMI—kg/m^2^	18 [18; 22]	22 [20; 26]	0.100
**COVID-19 vaccination**			
COVID-19 vaccinations before orthostatic symptom onset—n	2 [1; 2]	2 [1; 2]	0.476
Last vaccine type before orthostatic symptom onset—n (%)			0.226
Comirnaty	7 (78)	8 (80)	
Spikevax	2 (22)	0	
Vaxzevria	0	2 (20)	
Latency to orthostatic symptom onset—weeks	0 [0; 3]	0 [0; 0]	0.149
COVID-19 vaccinations—total n	2 [2; 2]	3 [2; 3]	0.183
Vaccine types overall—n (%)			
Comirnaty	6 (60)	8 (80)	0.471
Spikevax	2 (20)	0	
Vaxzevria	0	1 (10)	
Cross-vaccination	2 (20)	1 (10)	
**Additional post-vaccination autonomic complaints**			
Additional autonomic complaints—n (%)	10 (100)	5 (56)	0.033
Thermoregulatory/sudomotor—n (%)	10 (100)	2 (25)	0.002*
Respiratory—n (%)	0	1 (13)	1.000
Gastrointestinal—n (%)	7 (78)	2 (22)	0.057
Vasomotor—n (%)	2 (40)	1 (17)	0.545
Urogenital—n (%)	1 (13)	0	1.000
**Additional post-vaccination non-autonomic complaints and abnormalities**			
Additional non-autonomic complaints/abnormalities—n (%)	11 (100)	10 (100)	-
Additional non-autonomic complaints/abnormalities—n	5 [4; 7]	3 [2; 4]	0.028
Fatigue—n (%)	9 (100)	7 (88)	0.471
Neurocognitive—n (%)	6 (75)	3 (38)	0.315
Psychiatric—n (%)	5 (46)	3 (30)	0.659
Headache—n (%)	5 (63)	6 (67)	1.000
Sleep—n (%)	3 (38)	1 (17)	0.580
Cardiac—n (%)	6 (60)	3 (30)	0.370
Pulmonary—n (%)	7 (78)	2 (22)	0.057
Olfactory—n (%)	1 (33)	1 (25)	1.000
Neuromuscular—n (%)	1 (14)	1 (20)	1.000
Gustatory—n (%)	0	1 (25)	1.000
Other—n (%)	8 (80)	2 (22)	0.023
Laboratory—n (%)	8 (89)	5 (50)	0.141
**Comorbidities**			
Comorbidities—n (%)	6 (55)	8 (80)	0.361
Comorbidities—n	1 [0; 3]	2 [1; 4]	0.670
**Treatment**			
Behavioral measures—n (%)	11 (100)	NA	NA
Non-pharmacological measures—n (%)	11 (100)	NA	NA
Pharmacological measures—n (%)	6 (55)	NA	NA
**Follow-up**			
Available—n (%)	9 (82)	7 (70)	0.635
Follow-up time—months	7 [4; 15]	7 [3; 14]	0.967
Improved symptomatic course—n (%)	7 (78)	5 (71)	1.000

Quantitative values are shown as mean ± SD or median [25^th^, 75^th^ percentile]; qualitative variables as n (%). Distribution has been assessed with the Shapiro-Wilk test. Bold font indicates significant p-values after blockwise Bonferroni correction (* p<0.008). Data of n = 2 VVS (28 years old woman, 43 years old man) and n = 3 tOH (34 years old woman, 60 and 78 years old men) cases are not shown in the table above due to the low sample size preventing any meaningful statistical description. All received either the Comirnaty or Vaxzevria vaccine before their orthostatic symptom onset; at the last follow-up (available in n = 1 VVS, n = 2 tOH), one tOH case (who received pharmacotherapy) reported a symptomatic improvement. Likewise, due to limited data availability, additional autonomic function assessment data is shown for newly diagnosed POTS only (see Table 3). Abbreviations: CAD, cardiovascular autonomic disorder; COVID-19, coronavirus disease 2019; NA, not applicable; N/n, number; OIw/oCAD, orthostatic intolerance without CAD; POTS, postural orthostatic tachycardia syndrome; tOH, transient orthostatic hypotension; VVS, vasovagal syncope.

### Focus on newly diagnosed POTS following COVID-19 infection and vaccination

Table 3 shows the data and comparison of post-COVID versus post-vaccination newly diagnosed POTS cases, without significant differences observed.

**Table 3:** Newly diagnosed POTS following COVID-19 infection and vaccination

	POTS			*p*
Newly diagnosed	Total (N = 33)	Post-COVID (N = 22)	Post-vaccination (N = 11)	Post-COVID vs. Post-vaccination
**Demographics**				
Age—years	33 ± 9	33 ± 8	32 ± 10	0.612
Female sex—n (%)	27 (82)	19 (86)	8 (73)	0.375
Height—cm	167 ± 6	166 ± 7	169 ± 4	0.057
Weight—kg	59 ± 9	60 ± 10	55 ± 7	0.299
BMI—kg/m^2^	22 [18; 23]	22 [19; 24]	18 [18; 22]	0.131
**COVID-19**				
Latency to orthostatic symptom onset since COVID-19 infection or vaccination—weeks	0 [0; 3]	0 [0; 3]	0 [0; 3]	0.711
COVID-19 vaccinations—total n	2 [2; 3]	3 [2; 3]	2 [2; 2]	0.021
Vaccine types overall—n (%)				0.699
Comirnaty	18 (64)	12 (67)	6 (60)	
Spikevax	3 (11)	1 (6)	2 (20)	
Cross-vaccination	7 (25)	5 (28)	2 (20)	
**Additional post-COVID or post-vaccination autonomic complaints**				
Additional autonomic complaints—n (%)	29 (100)	19 (100)	10 (100)	–
Thermoregulatory/sudomotor—n (%)	20 (69)	10 (53)	10 (100)	0.011
Respiratory—n (%)	6 (23)	6 (33)	0	0.132
Gastrointestinal—n (%)	13 (45)	6 (30)	7 (78)	0.041
Vasomotor—n (%)	6 (29)	4 (25)	2 (40)	0.598
Urogenital—n (%)	3 (12)	2 (11)	1 (13)	1.000
**Additional post-COVID or post-vaccination non-autonomic complaints and abnormalities**				
Additional non-autonomic complaints/abnormalities—n (%)	33 (100)	22 (100)	11 (100)	–
Additional non-autonomic complaints/abnormalities –n	5 [3; 7]	5 [3; 7]	5 [4; 7]	0.564
Fatigue—n (%)	28 (97)	19 (95)	9 (100)	1.000
Neurocognitive—n (%)	22 (76)	16 (76)	6 (75)	1.000
Psychiatric—n (%)	10 (30)	5 (23)	5 (46)	0.240
Headache—n (%)	18 (69)	13 (72)	5 (63)	0.667
Sleep—n (%)	15 (68)	12 (86)	3 (38)	0.052
Cardiac—n (%)	10 (39)	4 (25)	6 (60)	0.109
Pulmonary—n (%)	14 (54)	7 (41)	7 (78)	0.110
Olfactory—n (%)	5 (31)	4 (31)	1 (33)	1.000
Neuromuscular—n (%)	5 (22)	4 (25)	1 (14)	1.000
Gustatory—n (%)	3 (20)	3 (25)	0	1.000
Other—n (%)	13 (57)	5 (39)	8 (80)	0.090
Laboratory—n (%)	24 (82)	16 (80)	8 (89)	1.000
**Comorbidities**				
Comorbidities—n (%)	23 (70)	17 (77)	6 (55)	0.240
Comorbidities—n	2 [0; 3]	2 [1; 3]	1 [0; 3]	0.701
Neurologic—n (%)	15 (46)	11 (50)	4 (36)	0.712
Migraine—n (%)	11 (73)	7 (64)	4 (100)	-
Psychiatric—n (%)	8 (24)	6 (27)	2 (18)	0.687
Cardiac—n (%)	3 (9)	2 (9)	1 (9)	1.000
Pulmonary—n (%)	6 (18)	5 (23)	1 (9)	0.637
Asthma—n (%)	4 (67)	3 (60)	1 (100)	–
Metabolic/endocrine—n (%)	5 (15)	3 (14)	2 (18)	1.000
Autoimmune/rheumatic—n (%)	3 (9)	1 (5)	2 (18)	0.252
Dermatologic—n (%)	3 (9)	2 (9)	1 (9)	1.000
Gastrointestinal—n (%)	3 (9)	1 (5)	2 (18)	0.252
Urologic—n (%)	2 (6)	2 (9)	0	0.542
Gynecologic—n (%)	2 (6)	1 (5)	1 (9)	1.000
Orthopedic—n (%)	3 (9)	3 (14)	0	0.534
Other—n (%)	6 (18)	3 (14)	3 (27)	0.375
**Treatment**				
Behavioral measures—n (%)	33 (100)	22 (100)	11 (100)	–
Non-pharmacological measures—n (%)	33 (100)	22 (100)	11 (100)	–
Pharmacological measures—n (%)	12 (36)	6 (27)	6 (55)	0.149
**Additional autonomic function assessments**				
Pathological 24 h ABPM—n (%)	1 (33)	0	1 (100)	0.333
Pathological QSART—n (%)	7 (78)	3 (75)	4 (80)	1.000
CASS - sudomotor dimension—n	2 [1; 2]	2 [0; 2]	2 [1; 2]	0.815
Initial orthostatic hypotension—n (%)	4 (14)	4 (22)	0	0.265
Delayed BP recovery—n (%)	1 (4)	1 (6)	0	1.000
Missing phase-II late BP overshoot on VM—n (%)	2 (7)	2 (11)	0	0.532
Missing phase-IV BP overshoot on VM—n (%)	1 (3)	1 (5)	0	1.000
CASS - cardiovagal dimension—n	0 [0; 0]	0 [0; 0]	0 [0; 0]	0.532
**Follow-up**				
Available—n (%)	28 (85)	19 (86)	9 (82)	1.000
Follow-up time—months	7 [4; 10]	7 [4; 10]	7 [4; 15]	0.280
Improved symptomatic course—n (%)	20 (71)	13 (68)	7 (78)	1.000
Postural orthostatic tachycardia on active standing test—n (%)	5 (50)	4 (50)	1 (50)	1.000

Quantitative values are shown as mean ± SD or median [25^th^, 75^th^ percentile]; qualitative variables as n (%). Distribution has been assessed with the Shapiro-Wilk test.

Abbreviations: BP, blood pressure; CASS, composite autonomic severity score; COVID-19, coronavirus disease 2019; N/n, number; POTS, postural orthostatic tachycardia syndrome; QSART, quantitative sudomotor axon reflex test; VM, Valsalva maneuver; 24-h ABPM, 24-h ambulatory blood pressure monitoring.

On QSART, most newly diagnosed POTS showed pathological findings (75% post-COVID vs. 80% post-vaccination; *p* = 1.000), with a median sudomotor CASS of 2 [1; 2] across both groups, i.e., on average accounting for moderate sudomotor dysfunction. Transient OH was rare and observed exclusively in post-COVID POTS individuals (i.e., in < 15% of newly diagnosed POTS overall). Likewise, a pathological BP counter-regulation during the Valsalva maneuver was rarely observed, nor did we observe any cardiovagal autonomic impairment across all newly diagnosed POTS (median cardiovagal CASS of 0 [0; 0]).

**Fig. 2:**
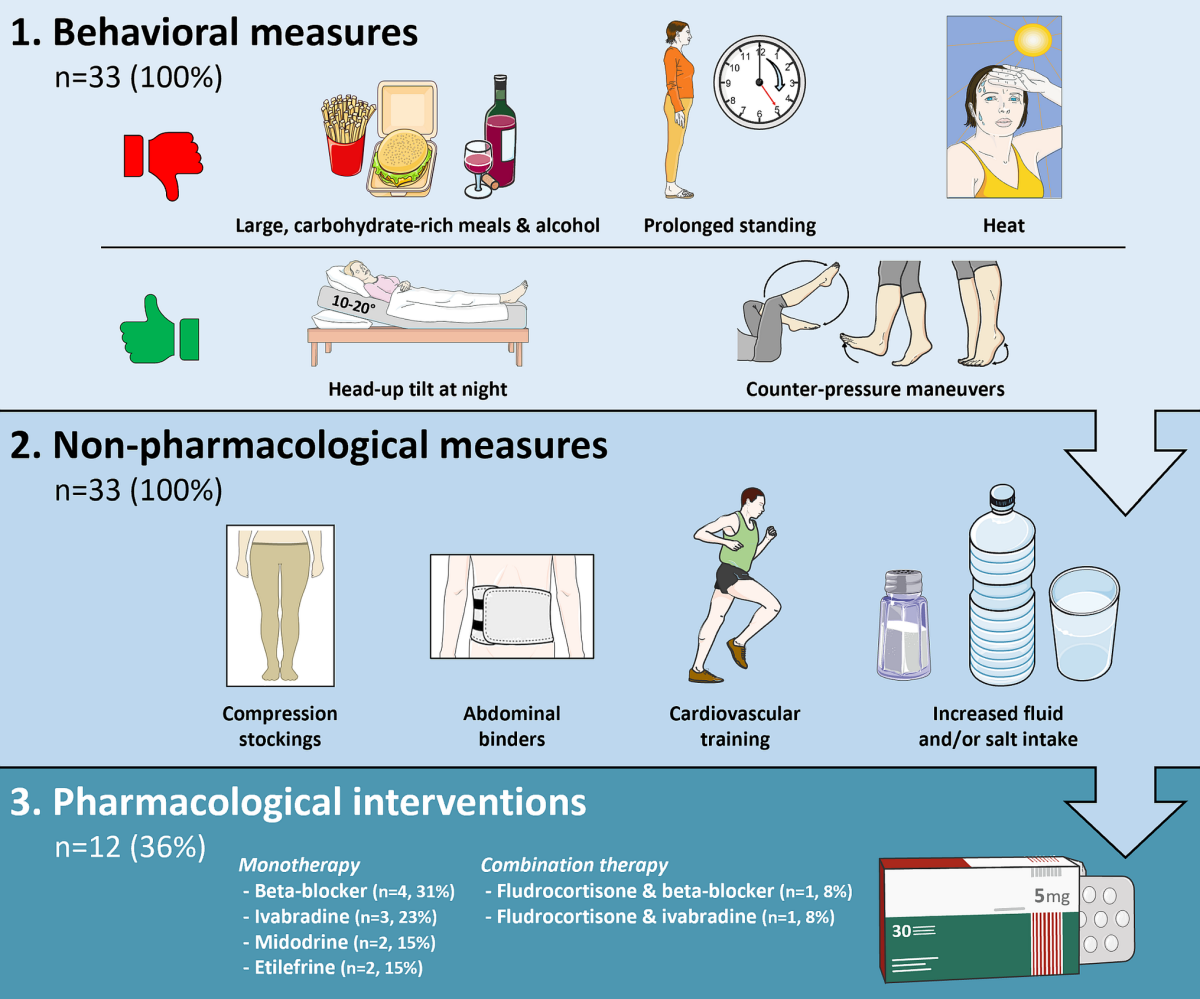
Treatment of newly diagnosed POTS following COVID-19 infection and vaccination. Created with Microsoft Office PowerPoint 2016, in part using adapted images provided by Servier Medical Art (https://smart.servier.com/), licensed under CC BY 4.0 (https://creativecommons.org/licenses/by/4.0/)

Twelve newly diagnosed POTS (*n* = 6 post-COVID vs. *n* = 6 post-vaccination; *p* = 0.149) received pharmacological treatment, either after the initial assessment or during follow-up, with one woman receiving two different pharmacological agents at different time points and two individuals initiated upon diagnosis on a combination therapy (comprising fludrocortisone plus either beta-blockers or ivabradine). Figure 2 illustrates the applied, stepwise therapeutic approach including the prescribed pharmacological agents.

Follow-up was available in 28 (85%) of newly diagnosed POTS, with a comparable follow-up duration (7 [4; 10] vs. 7 [4; 15] months; *p* = 0.280) and symptomatic improvement (68 vs. 78%; *p* = 1.000) across post-COVID and post-vaccination cases, respectively. Full recovery was observed in two post-COVID and one post-vaccination POTS case.

When comparing newly diagnosed POTS cases with versus without symptomatic improvement at follow-up, we did not observe any significant differences in the baseline characteristics (Supplementary Table 3). Non-improved POTS (*n* = 4, 100%) uniformly exhibited persistent postural tachycardia on the follow-up standing tests, whereas this was observed in one improved POTS case only (17%).

#### Post-COVID POTS case vignette

A 28-year-old woman with a history of migraine and previous vaccination against COVID-19 developed marked, persistent symptoms of OI within 2 weeks following a mild COVID-19 infection. She also reported intermittent, vague, burning-like pain in the upper extremities, discolored hands upon cold exposure, increased urinary frequency, diarrhea and abdominal bloating, as well as excessive sweating of the trunk. She contemporarily developed numerous additional non-autonomic complaints (*n* = 10), including fatigue, neurocognitive disturbances (brain fog and reduced concentration), and increase in frequency and intensity of her migraine episodes.

**Fig. 3:**
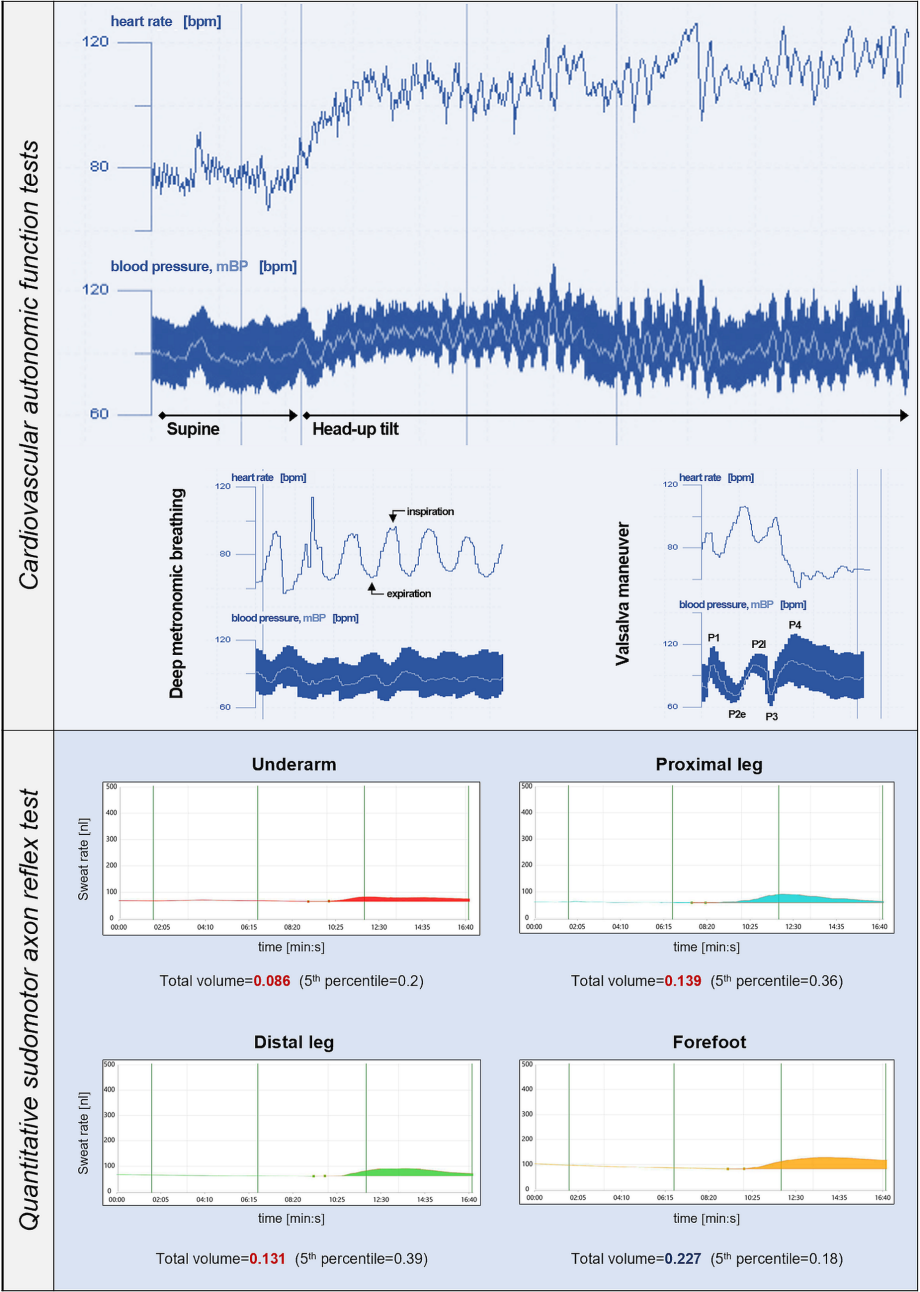
Post-COVID POTS case vignette. Valsalva maneuver phases: P1, onset of strain (increase of intrathoracic pressure by inspiration, causing a transient rise in BP due to blood being forced out of the chest cavity); P2, continued strain (early: reduced venous return to the heart due to elevated intrathoracic pressure causing a decrease in stroke volume and BP; late: vagal withdrawal and increased sympathetic discharge result in a heart rate increase and vasoconstriction to maintain BP); P3, release of strain (upon release of forced expiration, decrease of intrathoracic pressure resulting in a temporary fall in BP due to sudden increase in venous return and blood pooling in the pulmonary circulation); P4, recovery (BP overshoot in comparison to baseline levels as blood rushes back into the heart while peripheral resistance remains high from vasoconstriction, further triggering the baroreflex, which slows the heart rate and eventually returns BP to baseline). Created with Microsoft Office PowerPoint 2016

Except for cold hands, her neurological examination was normal. Laboratory analysis remained unremarkable, except for elevated calprotectin as well as positive S1-RBD- and nucleocapsid IgG. Tilt table testing disclosed postural orthostatic tachycardia with preserved BP counter regulation, while the Valsalva maneuver and deep metronomic breathing remained unremarkable. QSART revealed a diffuse pattern of postganglionic, sympathetic-cholinergic sudomotor dysfunction, and the patient was therefore diagnosed with *neuropathic POTS* [42–44]. Figure 3 illustrates the respective CAFT and QSART findings.

She was started on behavioral and further non-pharmacological measures (including compression stockings), which resulted in a marked symptomatic improvement within 7 months from the diagnosis. Ancillary findings and medical history indicate that the COVID-19 infection was associated with a widespread, yet reversible autonomic dysfunction in this case.

### Exacerbated CAD following COVID-19 infection and vaccination

Following COVID-19 infection, a pronounced clinical exacerbation of previously diagnosed CAD was recorded in 15 cases (i.e., in 6 POTS [40%], 6 VVS [40%], 1 classic [7%], and 2 transient [13%] OH). Following COVID-19 vaccination, we recorded four previously diagnosed CAD (i.e., 2 VVS, 1 delayed, and 1 transient OH case) with a marked symptomatic exacerbation (Supplementary Fig. 2). We observed no difference in the clinical-demographic characteristics of these cases (Supplementary Tables 4 and 5).

## Discussion

Back in 2020, the Region of Tyrol emerged as an early epidemiological hotspot of the COVID-19 pandemic [45]. Following federal decree, Austria entered its first lockdown on March 14, 2020, reducing social life and non-emergency medical service to the necessary minimum. Managing a growing number of individuals with post-COVID-19 disturbances amid limited understanding of the disease mechanisms [46] posed an unprecedented challenge early on. The Innsbruck Dysautonomia Center responded swiftly, adapting safety protocols and resuming activities within 6 weeks. Since then, through the Tyrol-wide *post-COVID program* (https://www.postcovid.tirol/) and national tertiary referrals, a substantial number of individuals with new-onset OI or recurrent syncope following COVID-19 infection and vaccination have been admitted, evaluated, and treated in our center.

Consistent with previous reports [26], POTS was the most frequent newly diagnosed CAD following COVID-19 in our cohort, in about one-third of referred cases, and exceeded pre-pandemic rates of 5% (2–15%) reported by European autonomic laboratories [20, 47]. Infectious illnesses are well-recognized POTS triggers, with up to 40% of COVID-unrelated POTS cases reporting a post-infectious symptom onset (e.g., following Epstein–Barr virus, *Mycoplasma pneumoniae*, influenza, or other upper respiratory infections) [20, 48]. Although growing evidence [2, 24, 26, 49–51] indicates an increased POTS prevalence in the post-COVID era, it remains a matter of debate whether this apparent rise reflects a true increase or results from greater awareness, intensified diagnostic testing, or selection and referral biases.

The prevalence of VVS in our post-COVID cohort matched pooled pre-pandemic estimates [22]. Reflex syncope and BP instability during or after an acute SARS-CoV-2 infection may result from fever- or cytokine-induced hypovolemia and vasodilation [11]. The comparable pre- to pandemic rates suggest an unmasking of pre-existing susceptibility to VVS rather than a SARS-CoV-2-specific mechanism of disease.

We observed no OH cases, and initial OH was less frequent than in a comparable Canadian post-COVID cohort (8% vs. 61%) [50], likely reflecting our more stringent temporal criterion.

Most newly diagnosed or worsened CAD occurred after COVID-19 infection. Given regional COVID-19 infection and vaccination rates [52–55], our data suggest that a COVID-19 infection poses a greater risk for developing or aggravating CAD than COVID-19 vaccination.

Previous evidence suggests that autonomic dysfunction after COVID-19 vaccination is in fact rare and causality unlikely [28–31]. A recent study found, however, that vaccination against COVID-19 can predispose individuals to develop small fiber neuropathy [56]. Small fiber neuropathy is regarded as one of the potential mechanisms underlying the pathophysiology of POTS [20], and in our cohort, QSART abnormalities indicating postganglionic small fiber dysfunction occurred with similar frequency in post-COVID and post-vaccination POTS. While post-vaccination cardiovascular autonomic dysfunction remains rare [32], small fiber neuropathy offers a plausible explanation for POTS cases manifesting after COVID-19 vaccination.

Newly diagnosed post-COVID POTS cases had the highest vaccination rate among all diagnostic groups, significantly exceeding that of post-COVID VVS. A post-hoc analysis indicated that this difference reflected timing, as most VVS diagnoses preceded vaccine availability in Austria. VVS was also the most frequently reported CAD during the acute COVID-19 phase [26], with syncope rates of up 12% in hospitalized COVID-19 individuals [57]. The observed decline in newly diagnosed VVS cases after introduction of COVID-19 vaccines, both in our cohort and in the literature [26], may reflect vaccination mitigating infection severity or the emergence of milder viral variants (e.g., Omicron). While vaccination reduces post-COVID sequelae overall [58], this may not apply to post-COVID POTS, as observed in our series.

Newly diagnosed POTS cases were younger than VVS and OIw/oCAD ones. Besides small fiber neuropathy, proposed POTS mechanisms include immune-mediated inflammation triggered by autoantigens or persistent viral particles [11, 20]. The short latency between infection and symptoms onset observed in most cases indicates a direct immune-mediated process, and the stronger immune responsiveness of younger individuals may predispose them to POTS development both after COVID-19 infection and vaccination.

In line with the multifaceted presentation of POTS [44, 48], additional autonomic complaints were more frequent in POTS than in OIw/oCAD. This broader autonomic involvement, supported by QSART findings [35], along with preserved cardiovagal function, suggests that postural tachycardia in post-COVID POTS may reflect a compensatory response to impaired peripheral circulatory control rather than a primary disease mechanism.

In this series, POTS cases showed similar rates of psychiatric comorbidities to non-COVID POTS cohorts [48], but many were newly diagnosed with psychiatric disorders after COVID-19 infection or vaccination. While studies have reported higher frequencies of depression and anxiety in POTS [59, 60], the overall lifetime prevalence of psychiatric comorbidities appears comparable to the general population [61]. Mental stress, whether arising from a psychiatric disorder or a chronic physical illness, is an acknowledged trigger of central sympathetic activation that may aggravate postural tachycardia [62].

Using a stepwise therapeutic approach, most newly diagnosed POTS and CAD cases showed a significant symptomatic improvement at follow-up. Symptom improvement was not associated with demographics, COVID-19 severity, vaccination status, additional complaints, or comorbidities. Other European studies including older post-COVID cases [63, 64] found that comorbidities and older age were associated with worse functional outcome [65, 66]. The greater resilience of the autonomic and immune systems of younger individuals may underlie the favorable recovery seen in our cohort.

Compared to previous reports [26], strengths and novelty elements of the present study include its large sample size, comprehensive, multidisciplinary diagnostic workup with detailed characterization of associated autonomic and non-autonomic complaints [46], and the follow-up outcome assessment. To our knowledge, this is the largest single-center cohort of individuals with newly diagnosed or worsened CAD following COVID-19 infection or vaccination, an area largely underinvestigated to date.

Limitations of this study comprise its retrospective and monocentric design. Non-systematic data collection limited the availability of second-line investigations, such as the QSART or 24-h ABPM, which were only analyzed in POTS cases for sample size reasons. In individuals with previously diagnosed CAD or severe symptoms, the CAFT battery was occasionally limited to an active standing test to reduce the test-related burden. Notwithstanding, the rigorous clinical records both at the Innsbruck Dysautonomia Center and other campus departments enabled detailed data acquisition in most evaluated cases.

Unlike the 3-month benchmark for defining the onset of a post-COVID condition [4], we limited the orthostatic symptom onset to within 6 weeks from COVID-19 infection or vaccination. This aligns with provisional definitions for post-COVID neurological disorders [41], and a meta-analysis on post-COVID autonomic disturbances [26], aiming to strengthen causal inference. While we recognize that this approach may have excluded certain cases, the typically very short, or sometimes absent, latency to CAD onset observed in our cohort, together with the systematic exclusion of alternative differential diagnoses, supports the validity of the 6-week criterion.

Concluding, a specialized diagnostic workup proved pivotal to diagnose or exclude CAD in individuals presenting with new-onset OI or recurrent syncope following COVID-19 infection or vaccination. This underscores the need for improving access to specialized autonomic healthcare across countries [47], as the therapeutic approach to CAD differs substantially from the management of their internal and psychiatric mimicries. Younger individuals appear to be at increased risk for developing post-COVID autonomic disturbances, particularly POTS. At the same time, younger age may provide this population with a better functional capacity to recover. Irrespective of the presence of additional autonomic and non-autonomic complaints or comorbidities, a multimodal treatment approach with a stepwise introduction of non-pharmacological and pharmacological measures contributes to symptomatic improvement in a substantial number of individuals. Lessons learned from the COVID-19 pandemic may also apply to autonomic disorders caused by other pathogens.

## Ethical approval

This research study was conducted retrospectively from data obtained for clinical purposes. The study was approved by the Innsbruck Ethical Committee (EC-No.: 1113/2023) and conducted in accordance with the ethical standards laid down in the 1964 Declaration of Helsinki and its later amendments, the directives of the Austrian Agency for Research Integrity, as well as the current European Data Protection Regulation. Given the retrospective nature, participant consent was not required. The manuscript was prepared in line with the STrengthening the Reporting of OBservational studies in Epidemiology (STROBE) Statement version 4 (see Supplementary material).

## Supplementary Information

Below is the link to the electronic supplementary material.

Supplementary file1 (PDF 831 KB)

## References

[1] Raj SR, Arnold AC, Barboi A, Claydon VE, Limberg JK, Lucci VM et al (2021) Long-COVID postural tachycardia syndrome: an American Autonomic Society statement. Clin Auton Res 31(3):365–368. 10.1007/s10286-021-00798-2 33740207 10.1007/s10286-021-00798-2 PMC7976723

[2] Fanciulli A, Leys F, Krbot Skorić M, Carneiro DR, Calandra-Buonaura G, Camaradou J et al (2023) Impact of the COVID-19 pandemic on clinical autonomic practice in Europe a survey of the European Academy of Neurology (EAN) and the European Federation of Autonomic Societies (EFAS). Eur J Neurol. 10.1111/ene.15787 36920252 10.1111/ene.15787

[3] Fanciulli A, Skorić MK, Leys F, Carneiro DR, Campese N, Calandra-Buonaura G et al (2023) EFAS/EAN survey on the influence of the COVID-19 pandemic on European clinical autonomic education and research. Clin Auton Res 33(6):777–790. 10.1007/s10286-023-00985-3 37792127 10.1007/s10286-023-00985-3 PMC10751256

[4] Soriano JB, Murthy S, Marshall JC, Relan P, Diaz JV (2022) A clinical case definition of post-COVID-19 condition by a Delphi consensus. Lancet Infect Dis 22(4):e102–e107. 10.1016/s1473-3099(21)00703-9 34951953 10.1016/S1473-3099(21)00703-9 PMC8691845

[5] Mao L, Jin H, Wang M, Hu Y, Chen S, He Q, Chang J, Hong C, Zhou Y, Wang D, Miao X, Li Y, Hu Bo (2020) Neurologic manifestations of hospitalized patients with coronavirus disease 2019 in Wuhan, China. JAMA Neurol 77(6):683–690. 10.1001/jamaneurol.2020.1127 32275288 10.1001/jamaneurol.2020.1127 PMC7149362

[6] Misra S, Kolappa K, Prasad M, Radhakrishnan D, Thakur KT, Solomon T et al (2021) Frequency of neurologic manifestations in COVID-19: a systematic review and meta-analysis. Neurology 97(23):e2269–e2281. 10.1212/wnl.0000000000012930 34635561 10.1212/WNL.0000000000012930 PMC8665434

[7] D V, Sharma A, Kumar A, Flora SJS (2021) Neurological manifestations in COVID-19 patients: a meta-analysis. ACS Chem Neurosci 12(15):2776–2797. 10.1021/acschemneuro.1c00353 34260855 10.1021/acschemneuro.1c00353

[8] Bisaccia G, Ricci F, Recce V, Serio A, Iannetti G, Chahal AA et al (2021) Post-acute sequelae of COVID-19 and cardiovascular autonomic dysfunction: what do we know? J Cardiovasc Dev Dis 8(11):156. 10.3390/jcdd8110156 34821709 10.3390/jcdd8110156 PMC8621226

[9] Carmona-Torre F, Mínguez-Olaondo A, López-Bravo A, Tijero B, Grozeva V, Walcker M et al (2022) Dysautonomia in COVID-19 patients: a narrative review on clinical course, diagnostic and therapeutic strategies. Front Neurol 13:886609. 10.3389/fneur.2022.886609 35720084 10.3389/fneur.2022.886609 PMC9198643

[10] Gibbons CH (2019) Basics of autonomic nervous system function. Handb Clin Neurol 160:407–418. 10.1016/b978-0-444-64032-1.00027-8 31277865 10.1016/B978-0-444-64032-1.00027-8

[11] Fedorowski A, Fanciulli A, Raj SR, Sheldon R, Shibao CA, Sutton R (2024) Cardiovascular autonomic dysfunction in post-COVID-19 syndrome: a major health-care burden. Nat Rev Cardiol 21(6):379–395. 10.1038/s41569-023-00962-3 38163814 10.1038/s41569-023-00962-3

[12] Scala I, Rizzo PA, Bellavia S, Brunetti V, Colò F, Broccolini A et al (2022) Autonomic dysfunction during acute SARS-CoV-2 infection: a systematic review. J Clin Med 11(13):3883. 10.3390/jcm11133883 35807167 10.3390/jcm11133883 PMC9267913

[13] COVID-19: Statement by the Autonomic Nervous System Disorders Scientific Panel. EANnews: European Academy of Neurology; 2020. [Available from: https://www.eanpages.org/2020/04/17/covid-19-statement-by-the-autonomic-nervous-system-disorders-scientific-panel/ (accessed June 20, 2025)]

[14] Buoite Stella A, Furlanis G, Frezza NA, Valentinotti R, Ajcevic M, Manganotti P (2021) Autonomic dysfunction in post-COVID patients with and witfhout neurological symptoms: a prospective multidomain observational study. J Neurol 269:587–596. 10.1007/s00415-021-10735-y 34386903 10.1007/s00415-021-10735-y PMC8359764

[15] Dani M, Dirksen A, Taraborrelli P, Torocastro M, Panagopoulos D, Sutton R, Lim PB (2021) Autonomic dysfunction in “long COVID”: rationale, physiology and management strategies. Clin Med (Lond) 21(1):e63–e67. 10.7861/clinmed.2020-0896 33243837 10.7861/clinmed.2020-0896 PMC7850225

[16] Larsen NW, Stiles LE, Miglis MG (2021) Preparing for the long-haul: autonomic complications of COVID-19. Auton Neurosci 235:102841. 10.1016/j.autneu.2021.102841 34265539 10.1016/j.autneu.2021.102841 PMC8254396

[17] Fedorowski A, Sutton R (2023) Autonomic dysfunction and postural orthostatic tachycardia syndrome in post-acute COVID-19 syndrome. Nat Rev Cardiol 20(5):281–282. 10.1038/s41569-023-00842-w 36732397 10.1038/s41569-023-00842-w PMC9893964

[18] Hassani M, Fathi Jouzdani A, Motarjem S, Ranjbar A, Khansari N (2021) How COVID-19 can cause autonomic dysfunctions and postural orthostatic syndrome? a review of mechanisms and evidence. Neurol Clin Neurosci 9(6):434–442. 10.1111/ncn3.12548 34909198 10.1111/ncn3.12548 PMC8661735

[19] Chadda KR, Blakey EE, Huang CL, Jeevaratnam K (2022) Long COVID-19 and postural orthostatic tachycardia syndrome- is dysautonomia to be blamed? Front Cardiovasc Med 9:860198. 10.3389/fcvm.2022.860198 35355961 10.3389/fcvm.2022.860198 PMC8959615

[20] Vernino S, Bourne KM, Stiles LE, Grubb BP, Fedorowski A, Stewart JM et al (2021) Postural orthostatic tachycardia syndrome (POTS): state of the science and clinical care from a 2019 National Institutes of Health Expert Consensus Meeting - Part 1. Auton Neurosci 235:102828. 10.1016/j.autneu.2021.102828 34144933 10.1016/j.autneu.2021.102828 PMC8455420

[21] Wu JS, Yang YC, Lu FH, Wu CH, Chang CJ (2008) Population-based study on the prevalence and correlates of orthostatic hypotension/hypertension and orthostatic dizziness. Hypertens Res 31(5):897–904. 10.1291/hypres.31.897 18712045 10.1291/hypres.31.897

[22] Salari N, Karimi Z, Hemmati M, Mohammadi A, Shohaimi S, Mohammadi M (2024) Global prevalence of vasovagal syncope: a systematic review and meta-analysis. Global Epidemiol 7:100136. 10.1016/j.gloepi.2024.100136 10.1016/j.gloepi.2024.100136 PMC10821537 38283939

[23] Goodman BP, Khoury JA, Blair JE, Grill MF (2021) COVID-19 dysautonomia. Front Neurol 12:624968. 10.3389/fneur.2021.624968 33927679 10.3389/fneur.2021.624968 PMC8076737

[24] Shouman K, Vanichkachorn G, Cheshire WP, Suarez MD, Shelly S, Lamotte GJ et al (2021) Autonomic dysfunction following COVID-19 infection: an early experience. Clin Auton Res 31(3):385–394. 10.1007/s10286-021-00803-8 33860871 10.1007/s10286-021-00803-8 PMC8050227

[25] Milovanovic B, Djajic V, Bajic D, Djokovic A, Krajnovic T et al (2021) Assessment of autonomic nervous system dysfunction in the early phase of infection with SARS-CoV-2 virus. Front Neurosci 15:640835. 10.3389/fnins.2021.640835 34234638 10.3389/fnins.2021.640835 PMC8256172

[26] Reis Carneiro D, Rocha I, Habek M, Helbok R, Sellner J, Struhal W et al (2023) Clinical presentation and management strategies of cardiovascular autonomic dysfunction following a COVID-19 infection - a systematic review. Eur J Neurol 30(5):1528–1539. 10.1111/ene.15714 36694382 10.1111/ene.15714

[27] Anaya JM, Rojas M, Salinas ML, Rodríguez Y, Roa G, Lozano M et al (2021) Post-COVID syndrome. a case series and comprehensive review. Autoimmun Rev 20(11):102947. 10.1016/j.autrev.2021.102947 34509649 10.1016/j.autrev.2021.102947 PMC8428988

[28] Koh JS, Hoe RHM, Yong MH, Chiew HJ, Goh Y, Yong KP et al (2021) Hospital-based observational study of neurological disorders in patients recently vaccinated with COVID-19 mRNA vaccines. J Neurol Sci 430:120030. 10.1016/j.jns.2021.120030 34688190 10.1016/j.jns.2021.120030 PMC8511874

[29] Reddy S, Arora M (2021) A case of postural orthostatic tachycardia syndrome secondary to the messenger RNA COVID-19 vaccine. Cureus 13(5):e14837. 10.7759/cureus.14837 33968543 10.7759/cureus.14837 PMC8101507

[30] Hermel M, Sweeney M, Abud E, Luskin K, Criado JP, Bonakdar R et al (2022) COVID-19 vaccination might induce postural orthostatic tachycardia syndrome: a case report. Vaccines (Basel) 10(7):991. 10.3390/vaccines10070991 35891154 10.3390/vaccines10070991 PMC9323926

[31] Eldokla AM, Numan MT (2022) Postural orthostatic tachycardia syndrome after mRNA COVID-19 vaccine. Clin Auton Res 32(4):307–311. 10.1007/s10286-022-00880-3 35870086 10.1007/s10286-022-00880-3 PMC9308031

[32] Blitshteyn S, Fedorowski A (2022) The risks of POTS after COVID-19 vaccination and SARS-CoV-2 infection: more studies are needed. Nat Cardiovasc Res 1(12):1119–1120. 10.1038/s44161-022-00180-z 39196162 10.1038/s44161-022-00180-z

[33] Kwan AC, Ebinger JE, Wei J, Le CN, Oft JR, Zabner R et al (2022) Apparent risks of postural orthostatic tachycardia syndrome diagnoses after COVID-19 vaccination and SARS-Cov-2 infection. Nat Cardiovasc Res 1(12):1187–1194. 10.1038/s44161-022-00177-8 37303827 10.1038/s44161-022-00177-8 PMC10254901

[34] WHO Guidelines Approved by the Guidelines Review Committee. Clinical management of COVID-19: Living guideline. Geneva: World Health Organization © World Health Organization 2021; 2022

[35] Novak P (2011) Quantitative autonomic testing. J Vis Exp. 2011(53). 10.3791/2502 10.3791/2502 PMC3196175 21788940

[36] Fanciulli A, Strano S, Ndayisaba JP, Goebel G, Gioffrè L, Rizzo M et al (2014) Detecting nocturnal hypertension in Parkinson’s disease and multiple system atrophy: proposal of a decision-support algorithm. J Neurol 261(7):1291–1299. 10.1007/s00415-014-7339-2 24737171 10.1007/s00415-014-7339-2

[37] Fanciulli A, Campese N, Goebel G, Ndayisaba JP, Eschlboeck S, Kaindlstorfer C et al (2020) Association of transient orthostatic hypotension with falls and syncope in patients with Parkinson disease. Neurology 95(21):e2854–e2865. 10.1212/wnl.0000000000010749 32938788 10.1212/WNL.0000000000010749 PMC7734734

[38] Freeman R, Wieling W, Axelrod FB, Benditt DG, Benarroch E, Biaggioni I et al (2011) Consensus statement on the definition of orthostatic hypotension, neurally mediated syncope and the postural tachycardia syndrome. Clin Auton Res 21(2):69–72. 10.1007/s10286-011-0119-5 21431947 10.1007/s10286-011-0119-5

[39] van Wijnen VK, Finucane C, Harms MPM, Nolan H, Freeman RL, Westerhof BE et al (2017) Noninvasive beat-to-beat finger arterial pressure monitoring during orthostasis: a comprehensive review of normal and abnormal responses at different ages. J Intern Med 282(6):468–483. 10.1111/joim.12636 28564488 10.1111/joim.12636

[40] Brignole M, Moya A, de Lange FJ, Deharo JC, Elliott PM, Fanciulli A et al (2018) 2018 ESC guidelines for the diagnosis and management of syncope. Eur Heart J 39(21):1883–1948. 10.1093/eurheartj/ehy037 29562304 10.1093/eurheartj/ehy037

[41] Ellul MA, Benjamin L, Singh B, Lant S, Michael BD, Easton A et al (2020) Neurological associations of COVID-19. Lancet Neurol 19(9):767–783. 10.1016/s1474-4422(20)30221-0 32622375 10.1016/S1474-4422(20)30221-0 PMC7332267

[42] Gibbons CH, Bonyhay I, Benson A, Wang N, Freeman R (2013) Structural and functional small fiber abnormalities in the neuropathic postural tachycardia syndrome. PLoS ONE 8(12):e84716. 10.1371/journal.pone.0084716 24386408 10.1371/journal.pone.0084716 PMC3874039

[43] Raj SR (2014) Highlights in clinical autonomic neurosciences: novel insights about vasovagal syncope and postural tachycardia syndrome from autonomic testing. Auton Neurosci 185:5–7. 10.1016/j.autneu.2014.07.002 25053240 10.1016/j.autneu.2014.07.002 PMC4165653

[44] Raj SR, Guzman JC, Harvey P, Richer L, Schondorf R, Seifer C et al (2020) Canadian Cardiovascular Society position statement on postural orthostatic tachycardia syndrome (POTS) and related disorders of chronic orthostatic intolerance. Can J Cardiol 36(3):357–372. 10.1016/j.cjca.2019.12.024 32145864 10.1016/j.cjca.2019.12.024

[45] Kreidl P, Schmid D, Maritschnik S, Richter L, Borena W, Genger JW et al (2020) Emergence of coronavirus disease 2019 (COVID-19) in Austria. Wien Klin Wochenschr 132(21–22):645–652. 10.1007/s00508-020-01723-9 32816114 10.1007/s00508-020-01723-9 PMC7439636

[46] Ewing AG, Joffe D, Blitshteyn S, Brooks AES, Wist J, Bar-Yam Y et al (2025) Long COVID clinical evaluation, research and impact on society: a global expert consensus. Ann Clin Microbiol Antimicrob 24(1):27. 10.1186/s12941-025-00793-9 40254579 10.1186/s12941-025-00793-9 PMC12010688

[47] Habek M, Leys F, Krbot Skorić M, Reis Carneiro D, Calandra-Buonaura G, Camaradou J et al (2022) Clinical autonomic nervous system laboratories in Europe: a joint survey of the European Academy of Neurology and the European Federation of Autonomic Societies: a joint survey of the European Academy of Neurology and the European Federation of Autonomic Societies. Eur J Neurol 29(12):3633–3646. 10.1111/ene.15538 36056590 10.1111/ene.15538 PMC9826284

[48] Shaw BH, Stiles LE, Bourne K, Green EA, Shibao CA, Okamoto LE et al (2019) The face of postural tachycardia syndrome - insights from a large cross-sectional online community-based survey. J Intern Med 286(4):438–448. 10.1111/joim.12895 30861229 10.1111/joim.12895 PMC6790699

[49] Björnson M, Wijnbladh K, Törnberg A, Svensson-Raskh A, Svensson A, Ståhlberg M et al (2025) Prevalence and clinical impact of postural orthostatic tachycardia syndrome in highly symptomatic long COVID. Circ Arrhythm Electrophysiol 18(10):e013629. 10.1161/CIRCEP.124.013629 41025260 10.1161/CIRCEP.124.013629

[50] Hira R, Baker JR, Siddiqui T, Ranada SI, Soroush A, Karalasingham K et al (2023) Objective hemodynamic cardiovascular autonomic abnormalities in post-acute sequelae of COVID-19. Can J Cardiol 39(6):767–775. 10.1016/j.cjca.2022.12.002 36509178 10.1016/j.cjca.2022.12.002 PMC9733966

[51] Blitshteyn S, Whitelaw S (2021) Postural orthostatic tachycardia syndrome (POTS) and other autonomic disorders after COVID-19 infection: a case series of 20 patients. Immunol Res 69(2):205–211. 10.1007/s12026-021-09185-5 33786700 10.1007/s12026-021-09185-5 PMC8009458

[52] Seekircher L, Siller A, Astl M, Tschiderer L, Wachter GA, Pfeifer B et al (2022) Seroprevalence of anti-SARS-CoV-2 IgG antibodies in Tyrol, Austria: updated analysis involving 22,607 blood donors covering the period October 2021 to April 2022. Viruses 14(9):1877. 10.3390/v14091877 36146684 10.3390/v14091877 PMC9502884

[53] Siller A, Seekircher L, Wachter GA, Astl M, Tschiderer L, Pfeifer B et al (2022) Seroprevalence, waning and correlates of anti-SARS-CoV-2 IgG antibodies in Tyrol, Austria: large-scale study of 35,193 blood donors conducted between June 2020 and September 2021. Viruses 14(3):568. 10.3390/v14030568 35336975 10.3390/v14030568 PMC8954543

[54] Siller A, Seekircher L, Astl M, Tschiderer L, Wachter GA, Penz J et al (2024) Anti-SARS-CoV-2 IgG seroprevalence in Tyrol, Austria, among 28,768 blood donors between May 2022 and March 2023. Vaccines. 10.3390/vaccines12030284 38543918 10.3390/vaccines12030284 PMC10976183

[55] Tschiderer L, Innerhofer H, Seekircher L, Waltle L, Richter L, Kimpel J et al (2024) Long-term effectiveness of an ultra-rapid rollout vaccination campaign with BNT162b2 on the incidence of SARS-CoV-2 infection. iScience 27(11):111117. 10.1016/j.isci.2024.111117 39555399 10.1016/j.isci.2024.111117 PMC11567098

[56] Donadio V, Incensi A, Furia A, Parisini S, Colaci F, Giannoccaro MP et al (2025) Small fiber neuropathy associated with COVID-19 infection and vaccination: a prospective case-control study. Eur J Neurol 32(1):e16538. 10.1111/ene.16538 39526678 10.1111/ene.16538 PMC11625946

[57] Chou SH, Beghi E, Helbok R, Moro E, Sampson J, Altamirano V et al (2021) Global incidence of neurological manifestations among patients hospitalized with COVID-19-a report for the GCS-NeuroCOVID Consortium and the ENERGY Consortium. JAMA Netw Open 4(5):e2112131. 10.1001/jamanetworkopen.2021.12131 33974053 10.1001/jamanetworkopen.2021.12131 PMC8114143

[58] Català M, Mercadé-Besora N, Kolde R, Trinh NTH, Roel E, Burn E et al (2024) The effectiveness of COVID-19 vaccines to prevent long COVID symptoms: staggered cohort study of data from the UK, Spain, and Estonia. Lancet Respir Med 12(3):225–236. 10.1016/s2213-2600(23)00414-9 38219763 10.1016/S2213-2600(23)00414-9

[59] Anderson JW, Lambert EA, Sari CI, Dawood T, Esler MD, Vaddadi G et al (2014) Cognitive function, health-related quality of life, and symptoms of depression and anxiety sensitivity are impaired in patients with the postural orthostatic tachycardia syndrome (POTS). Front Physiol 5:230. 10.3389/fphys.2014.00230 25009504 10.3389/fphys.2014.00230 PMC4070177

[60] Attard A, Attard S, Stanniland C, Iles A, Rajappan K, Moazami S et al (2023) Management of psychiatric conditions in patients with comorbid postural orthostatic tachycardia syndrome: a literature review and case vignette. Prim Care Companion CNS Disord 25(1):22nr03243. 10.4088/PCC.22nr03243 36763833 10.4088/PCC.22nr03243

[61] Raj V, Haman KL, Raj SR, Byrne D, Blakely RD, Biaggioni I et al (2009) Psychiatric profile and attention deficits in postural tachycardia syndrome. J Neurol Neurosurg Psychiatry 80(3):339–344. 10.1136/jnnp.2008.144360 18977825 10.1136/jnnp.2008.144360 PMC2758320

[62] Osei J, Vaccarino V, Wang M, Shah AS, Lampert R, Li LY et al (2024) Stress-induced autonomic dysfunction is associated with mental stress-induced myocardial ischemia in patients with coronary artery disease. Circ Cardiovasc Imag 17(6):e016596. 10.1161/circimaging.124.016596 10.1161/CIRCIMAGING.124.016596 PMC11187646 38868952

[63] Beghi E, Helbok R, Crean M, Chou SH, McNett M, Moro E et al (2021) The European Academy of Neurology COVID-19 registry (ENERGY): an international instrument for surveillance of neurological complications in patients with COVID-19. Eur J Neurol 28(10):3303–3323. 10.1111/ene.14652 33220127 10.1111/ene.14652 PMC7753513

[64] Cavallieri F, Sellner J, Akhvlediani T, Bassetti CL, Bereczki D, Fanciulli A et al (2025) The European Academy of Neurology NeuroCOVID-19 Task Force: a lesson for the future. Eur J Neurol 32(1):e16321. 10.1111/ene.16321 38676302 10.1111/ene.16321 PMC11618110

[65] Beghi E, Helbok R, Ozturk S, Karadas O, Lisnic V, Grosu O et al (2022) Short- and long-term outcome and predictors in an international cohort of patients with neuro-COVID-19. Eur J Neurol 29(6):1663–1684. 10.1111/ene.15293 35194889 10.1111/ene.15293 PMC9111799

[66] Jamal SM, Landers DB, Hollenberg SM, Turi ZG, Glotzer TV, Tancredi J et al (2022) Prospective evaluation of autonomic dysfunction in post-acute sequela of COVID-19. J Am Coll Cardiol 79(23):2325–2330. 10.1016/j.jacc.2022.03.357 35381331 10.1016/j.jacc.2022.03.357 PMC8976261

