# Supplementary material for: Postural orthostatic tachycardia syndrome is the most frequent cardiovascular autonomic disorder following COVID-19 infection or vaccination
Source: J Neurol. 2025 Nov 22;272(12):783. doi: 10.1007/s00415-025-13518-x (PMC12640326; doi:10.1007/s00415-025-13518-x)
Supplement: Supplementary file 1 — Supplementary file1 (PDF 831 KB) [file 415_2025_13518_MOESM1_ESM.pdf]

Supplementary Material

Postural orthostatic tachycardia syndrome is the most frequent cardiovascular autonomic disorders following COVID-19 infection or vaccination

Fabian Leys MD PhD[1], Mara Verginer[1], Elias Kirchler[1], Loraine Marino[1], Georg Goebel PhD[2], Nicole Campese MD PhD[1], Sabine Eschlböck MD[1,3], Susanne Duerr MD[1], Gregor Broessner MD[1], Atbin Djamshidian-Tehrani MD PhD[1], Anna Heidbreder MD[1,4], Birgit Högl MD[1], Maria-Sophie Rothmund-Grenier BSc BA MSc[5], Katharina Hüfner MD[5], Sarah Iglseder MD PhD[1], Wolfgang Löscher MD[1], Ambra Stefani MD PhD[1], Julia Wanschitz MD[1], Günter Weiss MD[6], Laura Zamarian MD PhD[1], Judith Löffler-Ragg MD[6], Raimund Helbok MD[1,4], Stefan Kiechl MD[1,7], Roberta Granata MD[1], Gregor K. Wenning MD PhD MSc[1ŧ] and Alessandra Fanciulli MD PhD[1]

[1]*Department of Neurology, Medical University of Innsbruck, Innsbruck, Austria*

[2]*Institute of Medical Statistics and Informatics, Medical University of Innsbruck, Innsbruck, Austria*

[3]*Department of Neurology, Hochzirl-Natters Hospital, Zirl, Austria*

[4]*Department of Neurology, Kepler University Clinic, Linz, Austria*

[5]*Department of Psychiatry, Psychotherapy, Psychosomatics and Medical Psychology, University Hospital of Psychiatry II, Medical University of Innsbruck, Innsbruck, Austria*

[6]*Department of Internal Medicine II, Medical University of Innsbruck, Innsbruck, Austria*

[7]*VASCage – Centre on Clinical Stroke Research, Innsbruck, Austria*

[ŧ]*Author deceased*

Corresponding author: Assoc.-Prof. Alessandra Fanciulli, MD PhD – Department of Neurology, Medical University of Innsbruck – E-Mail: alessandra.fanciulli@i-med.ac.at

Content

Supplementary Table 1. Definitions and variables ... 3

Supplementary Figure 1. Study flow chart ... 8

Supplementary Table 2. Improved versus not-improved CAD following COVID-19 infection ... 9

Supplementary Table 3. Improved versus not-improved, newly diagnosed POTS cases (overall and post-COVID) ... 11

Supplementary Figure 2. Diagnosis of individuals with exacerbated CAD following COVID-19 infection and vaccination ... 14

Supplementary Table 4. Exacerbated CAD following COVID-19 infection ... 15

Supplementary Table 5. Exacerbated CAD following COVID-19 vaccination ... 17

STrengthening the **R**eporting of **OB**servational studies in **E**pidemiology (**STROBE**) **Statement** version 4 ... 19

Supplementary Table 1. Definitions and variables

**Definitions**	
Postural orthostatic tachycardia syndrome (POTS)	Sustained HR increase of ≥30 bpm within 10 minutes of postural change (≥40 bpm required in individuals aged 12-19) without a fall in BP that qualifies for OH, accompanied by persistent symptoms of orthostatic intolerance for at least 3 months that quickly ameliorate upon sitting or lying down, in absence of other conditions/secondary factors promoting orthostatic sinus tachycardia, especially severe deconditioning caused by prolonged bed rest, ongoing infection or fever [1].
Vasovagal syncope	Neurally-/reflex-mediated sudden change in ANS activity with a fall of HR and/or BP resulting in transient loss of consciousness [2].
Classic orthostatic hypotension	Systolic and/or diastolic BP fall of ≥20/10 mmHg within three minutes of postural change [2].
Delayed orthostatic hypotension	Systolic and/or diastolic BP fall of ≥20/10 mmHg beyond three minutes of postural change [2].
Transient orthostatic hypotension	• <u>Initial OH:</u> profound systolic and/or diastolic BP fall of ≥40/20 mmHg occurring within 15 seconds of active standing, resolving within 30 seconds [2, 3].<br>• <u>Delayed BP recovery:</u> systolic BP fall of ≥20 mmHg after 30 seconds of postural change, with recovery within 1 minute [4].
Orthostatic intolerance without cardiovascular autonomic disorder (OIw/oCAD)	New-onset orthostatic intolerance without evidence of POTS, vasovagal syncope*, or any form of OH at cardiovascular autonomic function testing (* does not apply if history unambiguously indicates recurrent, reflex-mediated syncope).
Causal association of newly diagnosed CAD with COVID-19 infection	• <u>Probable:</u> orthostatic symptom onset within 6 weeks from an acute infection *AND* either SARS-CoV-2 RNA detected in any sample *OR* antibody evidence of acute SARS-CoV-2 infection *AND* no evidence of other commonly associated causes.<br>• <u>Possible:</u> orthostatic symptom onset within 6 weeks from an acute infection *AND* either SARS-CoV-2 RNA detected in any sample *OR* antibody evidence of acute SARS-CoV-2 infection *OR* clinical and epidemiological context of SARS-CoV-2 infection *AND* possibility of other commonly associated causes.
Causal association of newly diagnosed CAD with COVID-19 vaccination	• <u>Probable:</u> orthostatic symptom onset within 6 weeks from a vaccination against COVID-19 *AND* no evidence of other commonly associated causes.<br>• <u>Possible:</u> orthostatic symptom onset within 6 weeks from a vaccination against COVID-19 *AND* possibility of other commonly associated causes.

**Variables**	
Demographics	
Age	Age at the time of COVID-19 infection or causal vaccination in years.
Sex	Male or female.
Height	Height in cm.
Weight	Weight in kg.
Body mass index	Calculated as the weight in kg divided by height in square meters.
COVID-19 infection related	
Infection severity	Severity of COVID-19 infection [5]:<br>• asymptomatic: infection without occurrence of symptoms;<br>• mild: symptoms meeting the case definition for COVD-19 without evidence of viral pneumonia or hypoxia;<br>• moderate: clinical signs of pneumonia (fever, cough, dyspnea, fast breathing), but no signs of severe pneumonia, including oxygen saturation ≥90% on room air;<br>• severe: clinical signs of pneumonia (fever, cough, dyspnea) with either respiratory rate >30 breaths per minute, severe respiratory distress, or oxygen saturation <90% on room air;<br>• critical: clinical signs of acute respiratory distress syndrome, systemic inflammatory response syndrome, septic shock, acute thrombosis, or multisystem inflammatory syndrome in children.
Latency to orthostatic symptom onset/exacerbation	Latency to orthostatic symptom onset/exacerbation since COVID-19 infection in weeks.
Vaccinated at time of infection	Vaccinated at the time of COVID-19 infection (yes/no).
Number of COVID-19 vaccinations	If vaccinated, total number of COVID-19 vaccinations.

COVID-19 vaccination related	
Number of COVID-19 vaccinations before orthostatic symptom onset/exacerbation	Number of COVID-19 vaccinations before orthostatic symptom onset/exacerbation.
Last vaccine type before orthostatic symptom onset/exacerbation	Type of last vaccine received before orthostatic symptom onset/exacerbation (Comirnaty, Spikevax, Vaxzevria, Jcovden, Valneva, VidPrevtyn Beta).
Latency to orthostatic symptom onset/exacerbation	Latency to orthostatic symptom onset/exacerbation since COVID-19 vaccination in weeks.
Number of COVID-19 vaccinations	Total number of COVID-19 vaccinations.
Vaccine types overall	Types of COVID-19 vaccines received overall (Comirnaty, Spikevax, Vaxzevria, Jcovden, Valneva, VidPrevtyn Beta, and cross-vaccination).
Additional post-COVID or post-vaccination autonomic complaints	
Additional autonomic complaints	Presence of additional autonomic complaints (thermoregulatory/sudomotor, respiratory, gastrointestinal, vasomotor, urogenital; yes/no).
Additional post-COVID or post-vaccination non-autonomic complaints and abnormalities	
Additional non-autonomic complaints and abnormalities	Presence of additional non-autonomic complaints and abnormalities (fatigue, neurocognitive, psychiatric, headache, sleep, cardiac, pulmonary, olfactory, neuromuscular, gustatory, other, and laboratory; yes/no).
Number of additional non-autonomic complaints and abnormalities	Total number of additional non-autonomic complaints and/or abnormal findings.
Comorbidities at the time of COVID-19 infection or vaccination	
Comorbidities	In new-onset cases, presence of comorbidities (neurologic, psychiatric, cardiac, pulmonary, metabolic/endocrine, autoimmune/rheumatic, dermatologic, gastrointestinal, urologic, gynaecologic, orthopaedic, and other; yes/no).
Number of comorbidities	In new-onset cases, total number of comorbidities.

Applied treatment for autonomic complaints	
Behavioral measures	In newly diagnosed CAD, start of any behavioral measures (i.e., counter-pressure maneuvers, head-up tilt at night, trigger avoidance; yes/no).
Non-pharmacological measures	In newly diagnosed CAD, start of any non-pharmacological measures (i.e., increased fluid and/or salt intake, compression stockings, abdominal binders, cardiovascular training including physical rehabilitation [e.g. POTS-physiotherapy]; yes/no).
Pharmacological measures	In newly diagnosed CAD, start of any vasoactive pharmacological measures (including medications for HR control) after initial assessment or during follow-up (yes/no).
Add-on of non-pharmacological measures	In exacerbated CAD, start of any non-pharmacological measures (i.e., increased fluid and/or salt intake, compression stockings, abdominal binders, cardiovascular training including physical rehabilitation [e.g. POTS-physiotherapy]; yes/no).
Add-on of pharmacological measures	In exacerbated CAD, start of any vasoactive pharmacological measures (including medications for HR control) after initial assessment or during follow-up (yes/no).
Increase of pharmacological measures	In exacerbated CAD, increase of pre-existing vasoactive pharmacological measures (including medications for HR control; yes/no).
Additional autonomic function assessments	
Pathological 24h-ABPM	Evidence of hypotensive susceptibility, exercise- and postprandial hypotension, or disturbed circadian BP regulation on 24h-ABPM (yes/no) [3, 6-11].
Pathological QSART	Evidence of sudomotor dysfunction on quantitative sudomotor axon reflex test (yes/no) [12].
CASS – sudomotor dimension	Sudomotor CASS [12].
Initial orthostatic hypotension	Co-occurrence of initial OH [2, 3] in POTS, VVS, classic and delayed OH (yes/no).
Delayed BP recovery	Co-occurrence of delayed BP recovery [4] in POTS, VVS, classic and delayed OH (yes/no).
Missing phase-II late BP overshoot at Valsalva maneuver	Missing phase-II late systolic BP overshoot at Valsalva maneuver, as compared to phase-II early systolic BP levels (yes/no).

Missing phase-IV BP overshoot at Valsalva maneuver	Missing phase-IV systolic BP overshoot at Valsalva maneuver, as compared to phase-I systolic BP levels (yes/no).
CASS – cardiovagal dimension	Cardiovagal CASS [12].
Follow-up	
Availability of follow-up	At least one follow-up visit (yes/no).
Follow-up time	Follow-up time in months (calculated from the time of initial referral).
Improved symptomatic course	Significant improvement or full recovery of orthostatic intolerance and/or recurrent syncope at the time of last follow-up (yes/no).
Postural orthostatic tachycardia on active standing test	In newly diagnosed POTS, presence of postural orthostatic tachycardia (i.e., sustained HR increase of ≥30 bpm) on active standing test at the time of last follow-up (yes/no).

Abbreviations: BP, blood pressure; CAD, cardiovascular autonomic disorder; CASS, composite autonomic severity score; COVID-19, coronavirus disease 2019; HR, heart rate; OH, orthostatic hypotension; OIw/oCAD, orthostatic intolerance without CAD; POTS, postural orthostatic tachycardia syndrome; QSART, quantitative sudomotor axon reflex test; 24h-ABPM, 24-hour ambulatory BP monitoring.

Supplementary Figure 1. Study flow chart

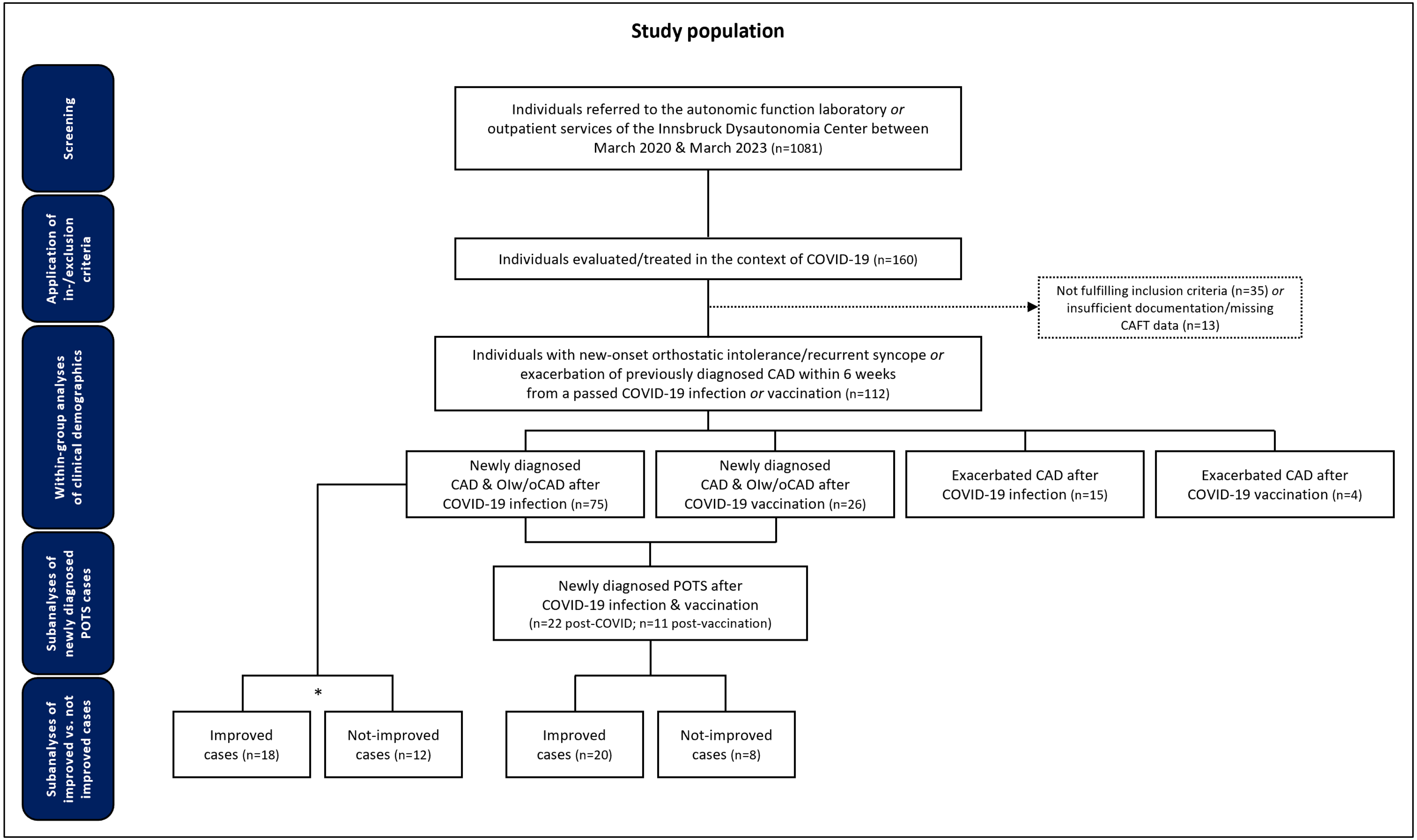

Asterisk indicates that OIw/oCAD were excluded from this subanalyses. Abbreviations: CAD, cardiovascular autonomic disorders; CAFT, cardiovascular autonomic function test; COVID-19, coronavirus disease 2019; n, number; OIw/oCAD, orthostatic intolerance without CAD; POTS, postural orthostatic tachycardia syndrome.

Supplementary Table 2. Improved versus not-improved CAD following COVID-19 infection

	Cardiovascular autonomic disorders			
**Newly diagnosed after COVID-19 infection**	**Followed-up N=30**	**Improved N=18**	**Not improved N=12**	***p***
**Demographics**				
Age – years	37±12	33±11	43±12	0.264
Female Sex – n (%)	25 (83)	15 (83)	10 (83)	1.000
Height – cm (n=12 improved; n=6 not-improved)	166±7	167±8	165±6	0.683
Weight – kg (n=12 improved; n=6 not-improved)	64±14	63±14	67±16	0.616
BMI – kg/m² (n=12 improved; n=6 not-improved)	22 [20; 24]	22 [20; 24]	24 [21; 27]	0.620
**Cardiovascular autonomic diagnoses**				
Type of CAD – n (%)				0.384
Postural orthostatic tachycardia syndrome	19 (63)	13 (72)	6 (50)	
Vasovagal syncope	9 (30)	5 (28)	4 (33)	
Delayed orthostatic hypotension	1 (3)	0	1 (8)	
Transient orthostatic hypotension	1 (3)	0	1 (8)	
**COVID-19 infection**				
Infection severity – n (%) (n=11 improved; n=11 not-improved)				1.000
Mild	10 (46)	5 (46)	5 (46)	
Moderate	10 (46)	5 (46)	5 (46)	
Severe	1 (5)	0	1 (9)	
Critical	1 (5)	1 (9)	0	
Latency to orthostatic symptom onset – weeks	0 [0; 2]	1 [0; 3]	0 [0; 1]	0.465
Vaccinated at time of infection – n (%) (n=16 improved)	17 (61)	9 (56)	8 (67)	0.705
COVID-19 vaccinations – total n (n=15 improved)	3 [2; 3]	3 [2; 3]	3 [2; 3]	0.188
**Additional post-COVID autonomic complaints**				
Additional autonomic complaints – n (%) (n=15 improved; n=9 not-improved)	22 (92)	15 (100)	7 (78)	0.130
Thermoregulatory/sudomotor – n (%) (n=17 improved; n=9 not-improved)	13 (50)	8 (47)	5 (56)	1.000
Respiratory – n (%) (n=12 improved; n=9 not-improved)	6 (29)	3 (25)	3 (33)	1.000
Gastrointestinal – n (%) (n=16 improved; n=10 not-improved)	8 (31)	4 (25)	4 (40)	0.664
Vasomotor – n (%) (n=12 improved; n=8 not-improved)	5 (25)	3 (25)	2 (25)	1.000
Urogenital – n (%) (n=15 improved; n=10 not-improved)	4 (16)	2 (13)	2 (20)	1.000
**Additional post-COVID non-autonomic complaints and abnormalities**				
Additional non-autonomic complaints/abnormalities – n (%)	30 (100)	18 (100)	12 (100)	-
Additional affected non-autonomic complaints/abnormalities – n	5 [3; 8]	5 [2; 8]	6 [4; 8]	0.457
Fatigue – n (%) (n=16 improved)	27 (96)	15 (94)	12 (100)	1.000
Neurocognitive – n (%) (n=17 improved)	21 (72)	12 (71)	9 (75)	1.000

Psychiatric – n (%)	9 (30)	5 (28)	3 (33)	1.000
Headache – n (%) (n=13 improved)	18 (72)	9 (69)	9 (75)	1.000
Sleep – n (%) (n=12 improved; n=10 not-improved)	17 (77)	9 (75)	8 (80)	1.000
Cardiac – n (%) (n=12 improved; n=11 not-improved)	5 (22)	2 (17)	3 (27)	0.640
Pulmonary – n (%) (n=15 improved; n=8 not-improved)	13 (57)	8 (53)	5 (63)	1.000
Olfactory – n (%) (n=12 improved; n=9 not-improved)	10 (48)	6 (50)	4 (44)	1.000
Neuromuscular – n (%) (n=12 improved; n=11 not-improved)	5 (22)	3 (25)	2 (18)	1.000
Gustatory – n (%) (n=10 improved; n=9 not-improved)	9 (47)	5 (50)	4 (44)	1.000
Other – n (%) (n=11 improved n=9 not-improved)	9 (45)	4 (36)	5 (56)	0.653
Laboratory abnormalities – n (%) (n=10 not-improved)	23 (82)	13 (72)	10 (100)	0.128
**Comorbidities**				
Comorbidities – n (%)	26 (87)	15 (83)	11 (92)	0.632
Comorbidities – n	2 [1; 3]	2 [1; 3]	3 [1; 4]	0.710
**Treatment**				
Behavioral measures – n (%) (n=17 improved)	29 (100)	17 (100)	12 (100)	-
Non-pharmacological measures – n (%) (n=17 improved)	29 (100)	17 (100)	12 (100)	-
Pharmacological measures – n (%) (n=17 improved)	8 (28)	4 (24)	4 (33)	0.683
**Follow-up**				
Follow-up-time – months	7 [4; 10]	7 [3; 10]	8 [4; 11]	0.626

Quantitative values are shown as mean±SD or median [25th, 75th percentile]; qualitative variables as n (%). Distribution has been assessed with the Shapiro-Wilk test.

Orthostatic intolerance without CAD (OIw/oCAD) cases were excluded from this analysis.

Abbreviations: CAD, cardiovascular autonomic disorders; COVID-19, coronavirus disease 2019; N/n, number.

Supplementary Table 3. Improved versus not-improved, newly diagnosed POTS cases (overall and post-COVID)

	Total POTS					Post-COVID POTS			
	Followed-up N=28	Improved N=20	Not improved N=8	*p*		Followed-up N=19	Improved N=13	Not improved N=6	*p*
**Demographics**									
Age – years	32±9	31±8	36±12	0.146	Age – years	33±9	30±7	38±11	0.099
Female Sex – n (%)	23 (82)	17 (85)	6 (75)	0.606	Female Sex – n (%)	16 (84)	11 (85)	5 (83)	1.000
Height – cm (n=13 improved; n=3 not-improved)	167±6	168±7	165±1	0.510	Height – cm (n=8 improved; n=2 not-improved)	166±8	167±8	165±1	1.000
Weight – kg (n=13 improved; n=3 not-improved)	57±7	57±8	58±7	0.750	Weight – kg (n=8 improved; n=2 not-improved)	58±8	59±8	56±6	1.000
BMI – kg/m² (n=13 improved; n=3 not-improved)	21 [18; 22]	20 [18; 22]	22 [19; -]	0.418	BMI – kg/m² (n=8 improved; n=2 not-improved)	22 [19; 22]	22 [19; 23]	21 [19; -]	1.000
**COVID-19**									
Latency to orthostatic symptom onset since COVID-19 infection or vaccination – weeks	0 [0; 3]	0 [0; 3]	1 [0; 4]	1.000	Latency to orthostatic symptom onset since COVID-19 infection – weeks	0 [0; 4]	1 [0; 4]	0 [0; 4]	0.628
COVID-19 vaccinations – total n (n=18 improved)	3 [2; 3]	2 [2; 3]	3 [2; 3]	0.202	COVID-19 vaccinations – total n (n=11 improved)	3 [2; 3]	3 [2; 3]	3 [3; 3]	0.353
Vaccine types overall – n (%) (n=17 improved)				0.473	Vaccine types overall – n (%) (n=11 improved)				0.744
Comirnaty	16 (64)	11 (65)	5 (63)		Comirnaty	12 (71)	8 (73)	4 (67)	
Spikevax	3 (12)	3 (18)	0		Spikevax	1 (6)	1 (9)	0	
Cross-vaccination	6 (24)	3 (18)	3 (38)		Cross-vaccination	4 (24)	2 (18)	2 (33)	
**Additional autonomic complaints**									
Additional autonomic complaints – n (%) (n=19 improved; n=6 not-improved)	25 (100)	19 (100)	6 (100)	-	Additional autonomic complaints – n (%) (n=12 improved; n=4 not-improved)	16 (100)	12 (100)	4 (100)	-
Thermoregulatory/sudomotor – n (%) (n=6 not-improved)	17 (65)	13 (65)	4 (67)	1.000	Thermoregulatory/sudomotor – n (%) (n=4 not-improved)	8 (47)	6 (46)	2 (50)	1.000
Respiratory – n (%) (n=17 improved; n=6 not-improved)	5 (22)	3 (18)	2 (33)	0.576	Respiratory – n (%) (n=11 improved; n=5 not-improved)	5 (31)	3 (27)	2 (40)	1.000
Gastrointestinal – n (%) (n=19 improved; n=6 not-improved)	11 (44)	9 (47)	2 (33)	0.661	Gastrointestinal – n (%) (n=12 improved; n=5 not-improved)	4 (24)	3 (25)	1 (20)	1.000
Vasomotor – n (%) (n=13 improved; n=5 not-improved)	5 (28)	4 (31)	1 (20)	1.000	Vasomotor – n (%) (n=10 improved; n=4 not-improved)	4 (29)	3 (30)	1 (25)	1.000
Urogenital – n (%) (n=17 improved; n=6 not-improved)	3 (13)	3 (18)	0	0.539	Urogenital – n (%) (n=11 improved; n=5 not-improved)	2 (13)	2 (18)	0	1.000

**Additional non-autonomic complaints and abnormalities**									
Additional non-autonomic complaints/abnormalities – n (%)	28 (100)	20 (100)	8 (100)	-	Additional non-autonomic complaints/abnormalities – n (%)	19 (100)	13 (100)	6 (100)	-
Additional non-autonomic complaints/abnormalities – n	5 [3; 7]	5 [3; 7]	6 [3; 7]	1.000	Additional non-autonomic complaints/abnormalities – n	5 [3; 7]	5 [3; 8]	5 [3; 6]	0.786
Fatigue – n (%) (n=17 improved)	24 (96)	16 (94)	8 (100)	1.000	Fatigue – n (%) (n=12 improved)	17 (94)	11 (92)	6 (100)	1.000
Neurocognitive – n (%) (n=18 improved)	19 (73)	14 (78)	5 (63)	0.635	Neurocognitive – n (%)	14 (74)	10 (77)	4 (67)	1.000
Psychiatric – n (%)	9 (32)	7 (35)	2 (25)	1.000	Psychiatric – n (%)	4 (21)	3 (23)	1 (17)	1.000
Headache – n (%) (n=17 improved; n=7 not-improved)	18 (75)	13 (77)	5 (71)	1.000	Headache – n (%) (n=11 improved)	13 (77)	8 (73)	5 (83)	1.000
Sleep – n (%) (n=15 improved; n=6 not-improved)	14 (67)	9 (60)	5 (83)	0.613	Sleep – n (%) (n=9 improved; n=4 not-improved)	11 (85)	7 (78)	4 (100)	1.000
Cardiac – n (%) (n=15 improved; n=7 not-improved)	7 (32)	4 (27)	3 (43)	0.630	Cardiac – n (%) (n=9 improved; n=5 not-improved)	3 (21)	2 (22)	1 (20)	1.000
Pulmonary – n (%) (n=18 improved; n=4 not-improved)	11 (50)	9 (50)	2 (50)	1.000	Pulmonary – n (%) (n=12 improved; n=3 not-improved)	6 (40)	5 (42)	1 (33)	1.000
Olfactory – n (%) (n=11 improved; n=4 not-improved)	5 (33)	4 (36)	1 (25)	1.000	Olfactory – n (%) (n=9 improved; n=3 not-improved)	4 (33)	4 (44)	0	0.491
Neuromuscular – n (%) (n=15 improved; n=7 not-improved)	5 (23)	4 (27)	1 (14)	1.000	Neuromuscular – n (%) (n=10 improved)	4 (25)	3 (30)	1 (17)	1.000
Gustatory – n (%) (n=10 improved; n=4 not-improved)	3 (21)	3 (30)	0	0.505	Gustatory – n (%) (n=8 improved; n=3 not-improved)	3 (27)	3 (38)	0	0.491
Other – n (%) (n=14 improved; n=6 not-improved)	12 (60)	8 (57)	4 (67)	1.000	Other – n (%) (n=8 improved; n=4 not-improved)	4 (33)	2 (25)	2 (50)	0.547
Laboratory – n (%) (n=19 improved; n=6 not-improved)	20 (80)	14 (74)	6 (100)	0.289	Laboratory – n (%) (n=4 not-improved)	13 (77)	9 (69)	4 (100)	0.519
**Comorbidities**									
Comorbidities – n (%)	21 (75)	15 (75)	6 (75)	1.000	Comorbidities – n (%)	15 (79)	10 (77)	5 (83)	1.000
Comorbidities – n	2 [0; 3]	2 [0; 3]	2 [0; 3]	0.758	Comorbidities – n	2 [1; 3]	2 [1; 3]	2 [1; 3]	0.984
Neurologic – n (%)	13 (46)	9 (45)	4 (50)	1.000	Neurologic – n (%)	9 (47)	6 (46)	3 (50)	1.000
Migraine – n (%)	9 (69)	6 (67)	3 (75)	-	Migraine – n (%)	5 (56)	3 (50)	2 (67)	-
Psychiatric – n (%)	7 (25)	4 (20)	3 (38)	0.371	Psychiatric – n (%)	5 (26)	3 (23)	2 (33)	1.000
Cardiac – n (%)	3 (11)	1 (5)	2 (25)	0.188	Cardiac – n (%)	2 (11)	0	2 (33)	0.088
Pulmonary – n (%)	6 (21)	4 (20)	2 (25)	1.000	Pulmonary – n (%)	5 (26)	4 (31)	1 (17)	1.000
Asthma – n (%)	4 (67)	2 (50)	2 (100)	-	Asthma – n (%)	3 (60)	2 (50)	1 (100)	-
Metabolic/endocrine – n (%)	4 (14)	4 (20)	0	0.295	Metabolic/endocrine – n (%)	2 (11)	2 (15)	0	1.000

Autoimmune/rheumatic – n (%)	3 (11)	3 (15)	0	0.536	Autoimmune/rheumatic – n (%)	1 (5)	1 (8)	0	1.000
Dermatologic – n (%)	3 (11)	2 (10)	1 (13)	1.000	Dermatologic – n (%)	2 (11)	1 (8)	1 (17)	1.000
Gastrointestinal – n (%)	3 (11)	1 (5)	2 (25)	0.188	Gastrointestinal – n (%)	1 (5)	0	1 (17)	0.316
Urologic – n (%)	2 (7)	2 (10)	0	1.000	Urologic – n (%)	2 (11)	2 (15)	0	1.000
Gynaecologic – n (%)	2 (7)	2 (10)	0	1.000	Gynaecologic – n (%)	1 (5)	1 (8)	0	1.000
Orthopaedic – n (%)	2 (7)	1 (5)	1 (13)	0.497	Orthopaedic – n (%)	2 (11)	1 (8)	1 (17)	1.000
Other – n (%)	5 (18)	5 (25)	0	0.281	Other – n (%)	2 (11)	2 (15)	0	1.000
**Treatment**									
Behavioral measures – n (%)	28 (100)	20 (100)	8 (100)	-	Behavioral measures – n (%)	19 (100)	13 (100)	6 (100)	-
Non-pharmacological measures – n (%)	28 (100)	20 (100)	8 (100)	-	Non-pharmacological measures – n (%)	19 (100)	13 (100)	6 (100)	-
Pharmacological measures – n (%)	11 (39)	6 (30)	5 (63)	0.200	Pharmacological measures – n (%)	6 (32)	3 (23)	3 (50)	0.320
**Additional autonomic function assessment**									
Pathological 24h-ABPM – n (%)<br>(n=3 improved)	1 (33)	1 (33)	-	-	Pathological 24h-ABPM – n (%)<br>(n=2 improved)	0	0	-	-
Pathological QSART – n (%)<br>(n=8 improved; n=1 not-improved)	7 (78)	6 (75)	1 (100)	1.000	Pathological QSART – n (%)<br>(n=3 improved; n=1 not-improved)	3 (75)	2 (67)	1 (100)	1.000
CASS – sudomotor dimension – n<br>(n=8 improved; n=1 not-improved)	2 [1; 2]	2 [0; 2]	2	-	CASS – sudomotor dimension – n<br>(n=3 improved; n=1 not-improved)	2 [0; 2]	1 [0; -]	2	-
Initial orthostatic hypotension – n (%)<br>(n=16 improved)	4 (17)	2 (13)	2 (25)	0.578	Initial orthostatic hypotension – n (%)<br>(n=9 improved)	4 (27)	2 (22)	2 (33)	1.000
Delayed BP recovery – n (%)<br>(n=16 improved)	1 (4)	0	1 (13)	0.333	Delayed BP recovery – n (%)<br>(n= 9 improved)	1 (7)	0	1 (17)	0.400
Missing phase-II late overshoot on VM – n (%)<br>(n=17 improved)	2 (8)	1 (6)	1 (13)	1.000	Missing phase-II late overshoot on VM – n (%)<br>(n=10 improved)	2 (13)	1 (10)	1 (17)	1.000
Missing phase-IV overshoot on VM – n (%)<br>(n=17 improved)	1 (4)	0	1 (13)	0.320	Missing phase-IV overshoot on VM – n (%)<br>(n=10 improved)	1 (6)	0	1 (17)	0.375
CASS – cardiovagal dimension – n<br>(n=17 improved)	0 [0; 0]	0 [0; 0]	0 [0; 0]	1.000	CASS- cardiovagal dimension – n<br>(n=10 improved)	0 [0; 0]	0 [0; 0]	0 [0; 0]	0.500
**Follow-up**									
Follow-up-time – months	7 [4; 10]	7 [3; 10]	9 [5; 13]	0.242	Follow-up-time – months	7 [4; 10]	7 [3; 10]	9 [4; 12]	0.258
Postural tachycardia on active standing test – n (%)<br>(n=6 improved; n=4 not-improved)	5 (50)	1 (17)	4 (100)	0.048	Postural tachycardia on active standing test – n (%)<br>(n=4 improved; n=4 not-improved)	4 (50)	0	4 (100)	0.029

Quantitative values are shown as mean±SD or median [25th, 75th percentile]; qualitative variables as n (%). Distribution has been assessed with the Shapiro-Wilk test. Given the available sample size, we did not compare improved versus not-improved post-vaccination POTS cases. Abbreviations: CASS, composite autonomic severity score; COVID-19, coronavirus disease 2019; N/n, number; POTS, postural orthostatic tachycardia syndrome; QSART, quantitative sudomotor axon reflex test; VM, Valsalva maneuver; 24h-ABPM, 24-hour ambulatory blood pressure monitoring.

Supplementary Figure 2. Diagnosis of individuals with exacerbated CAD following COVID-19 infection and vaccination

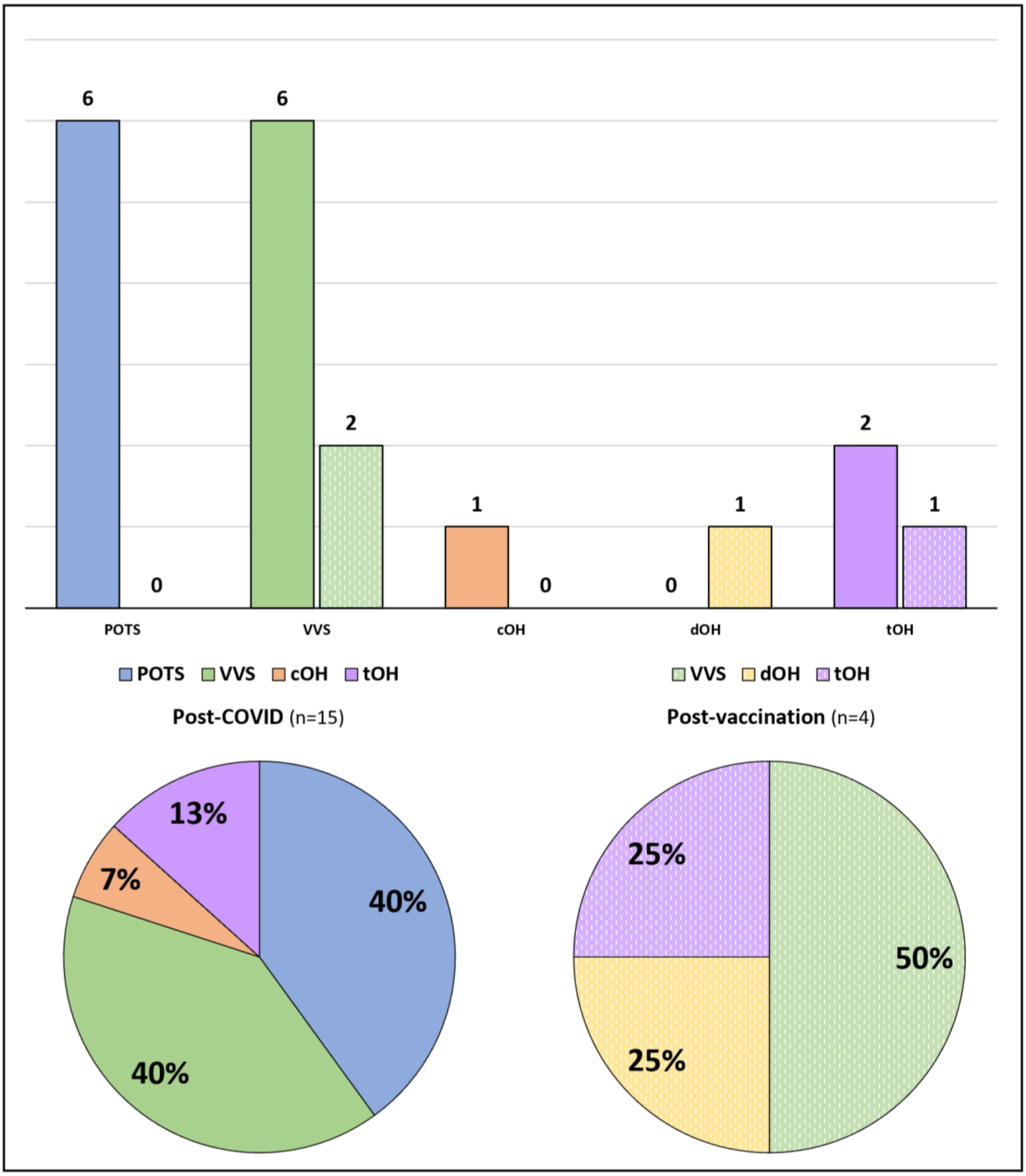

Abbreviations: CAD, cardiovascular autonomic disorders; cOH, classic orthostatic hypotension; COVID-19, coronavirus disease 2019; dOH, delayed OH; POTS, postural orthostatic tachycardia syndrome; tOH, transient OH; VVS, vasovagal syncope. Created with Microsoft Office PowerPoint 2016.

Supplementary Table 4. Exacerbated CAD following COVID-19 infection

	Cardiovascular autonomic disorders				*p*
**Exacerbated after COVID-19 infection**	**POTS N=6**	**VVS N=6**	**cOH N=1**	**tOH N=2**	**POTS vs. VVS**
**Demographics**					
Age – years	30±5	45±21	74	64±11	0.152
Female Sex – n (%)	6 (100)	6 (100)	0	0	-
Height – cm (n=3 VVS; n=1 tOH)	168±10	168±4	175	182	0.905
Weight – kg (n=3 VVS; n=1 tOH)	57±7	73±7	75	74	0.011
BMI – kg/m² (n=3 VVS; n=1 tOH)	21 [18; 22]	26 [22; -]	25	22	0.020
**COVID-19 infection**					
Infection severity – n (%) (n=3 POTS)					1.000
Asymptomatic	0	1 (17)	0	0	
Mild	2 (67)	4 (67)	1 (100)	1 (50)	
Moderate	1 (33)	1 (17)	0	0	
Severe	0	0	0	1 (50)	
Latency to orthostatic symptom exacerbation – weeks	0 [0; 3]	0 [0; 2]	0	2	1.000
Vaccinated at time of infection – n (%) (n=5 POTS)	3 (60)	3 (50)	0 (0)	1 (50)	1.000
COVID-19 vaccinations – total n (n=5 POTS; n=1 tOH)	2 [1; 3]	2 [1; 4]	-	2	1.000
**Additional post-COVID autonomic complaints**					
Additional autonomic complaints – n (%) (n=5 POTS; n=4 VVS)	5 (100)	3 (75)	-	1 (50)	0.444
Thermoregulatory/sudomotor – n (%) (n=5 VVS)	3 (50)	1 (20)	-	1 (50)	0.545
Respiratory – n (%) (n=5 POTS; n=4 VVS)	1 (20)	0	-	0	1.000
Gastrointestinal – n (%) (n=5 VVS)	3 (50)	2 (40)	-	1 (50)	1.000
Vasomotor – n (%) (n=3 POTS, n=5 VVS)	3 (100)	0	-	0	0.018
Urogenital – n (%) (n=4 VVS)	0	2 (50)	-	1 (50)	0.133
**Additional post-COVID non-autonomic complaints and abnormalities**					
Additional non-autonomic complaints/abnormalities – n (%)	6 (100)	6 (100)	1 (100)	2 (100)	-
Additional non-autonomic complaints/abnormalities – n	4 [2; 9]	4 [2; 6]	2	3 [2; -]	1.000
Fatigue – n (%) (n=4 VVS; n=1 tOH)	6 (100)	2 (50)	-	1 (100)	0.133
Neurocognitive – n (%) (n=3 POTS; n=2 VVS)	3 (100)	1 (50)	-	1 (50)	0.400
Psychiatric – n (%) (n=5 POTS)	1 (20)	2 (33)	0	1 (50)	1.000
Headache – n (%) (n=3 POTS; n=5 VVS)	2 (67)	3 (60)	-	1 (50)	1.000
Sleep – n (%) (n=2 POTS; n=4 VVS)	2 (100)	3 (75)	1 (100)	0	1.000
Cardiac – n (%) (n=5 VVS; n=1 tOH)	1 (17)	2 (40)	-	0	0.545

Pulmonary – n (%) (n=5 POTS; n=5 VVS; n=1 tOH)	2 (40)	2 (40)	-	0	1.000
Olfactory – n (%) (n=3 POTS; n=4 VVS)	3 (100)	2 (50)	-	0	0.429
Neuromuscular – n (%) (n=4 POTS; n=3 VVS; n=1 tOH)	1 (25)	0	-	1 (100)	1.000
Gustatory – n (%) (n=3 POTS; n=4 VVS)	3 (100)	2 (50)	-	0	0.429
Other – n (%) (n=5 POTS; n=5 VVS; n=1 tOH)	4 (80)	3 (60)	-	1 (100)	1.000
Laboratory – n (%) (n=5 POTS; n=5 VVS)	3 (60)	4 (80)	1 (100)	-	1.000
**Treatment**					
Add-on of non-pharmacological measures – n (%)	5 (83)	4 (67)	0	0	1.000
Add-on of pharmacological measures – n (%) (n=5 POTS)	1 (20)	3 (50)	0	1 (50)	0.545
Increase of pharmacological measures – n (%) (n=4 POTS; n=5 VVS, n=1 tOH)	0	0	1 (100)	0	-
**Follow-up**					
Available – n (%)	5 (83)	4 (67)	1 (100)	1 (50)	1.000
Follow-up-time – months (n=5 POTS; n=4 VVS; n=1 tOH)	7 [5; 21]	9 [6; 14]	22	7	0.716
Improved symptomatic course – n (%) (n=5 POTS; n=4 VVS; n=1 tOH)	4 (80)	2 (50)	-	1 (100)	0.524

Quantitative values are shown as mean±SD or median [25th, 75th percentile]; qualitative variables as n (%). Distribution has been assessed with the Shapiro-Wilk test. Abbreviations: CAD, cardiovascular autonomic disorders; cOH, classic orthostatic hypotension; COVID-19, coronavirus disease 2019; N/n, number; POTS, postural orthostatic tachycardia syndrome; tOH, transient OH; VVS, vasovagal syncope.

Supplementary Table 5. Exacerbated CAD following COVID-19 vaccination

	Cardiovascular autonomic disorders		
**Exacerbated after COVID-19 vaccination**	**VVS N=2**	**dOH N=1**	**tOH N=1**
**Demographics**			
Age – years	41±16	62	45
Female Sex – n (%)	2 (100)	0	1 (100)
**COVID-19 vaccination**			
COVID-19 vaccinations before orthostatic symptom exacerbation – n	2 [2; 2]	3	1
Last vaccine type before orthostatic symptom exacerbation – n (%)			
Comirnaty	2 (100)	1 (100)	0
Spikevax	0	0	1 (100)
Latency to orthostatic symptom exacerbation – weeks	2 [2; 2]	4	1
COVID-19 vaccinations – total n	4 [3; 0]	3	1
Vaccine types overall – n (%)			
Comirnaty	1 (50)	1 (100)	0
Spikevax	0	0	1 (100)
Cross-vaccination	1 (50)	0	0
**Additional post-vaccination autonomic complaints**			
Additional autonomic complaints – n (%) (n=1 VVS)	1 (100)	1 (100)	1 (100)
Thermoregulatory/sudomotor – n (%) (n=1 VVS)	1 (100)	0	-
Respiratory – n (%)	-	0	0
Gastrointestinal – n (%)	1 (50)	0	1 (100)
Vasomotor – n (%) (n=1 VVS)	0	0	1 (100)
Urogenital – n (%)	1 (50)	1 (100)	-
**Additional post-vaccination non-autonomic complaints and abnormalities**			
Additional non-autonomic complaints/abnormalities – n (%) (n=1 VVS)	1 (100)	1 (100)	1 (100)
Additional non-autonomic complaints/abnormalities – n (n=1 VVS)	8	2	5
Fatigue – n (%) (n=1 VVS)	1 (100)	-	1 (100)
Neurocognitive – n (%) (n=1 VVS)	1 (100)	-	0
Psychiatric – n (%)	1 (50)	0	0
Headache – n (%) (n=1 VVS)	1 (100)	0	-
Sleep – n (%) (n=1 VVS)	1 (100)	0	-
Cardiac – n (%)	0	-	1 (100)
Pulmonary – n (%) (n=1 VVS)	0	-	1 (100)
Olfactory – n (%) (n=1 VVS)	1 (100)	0	-
Neuromuscular – n (%)	-	-	-
Gustatory – n (%) (n=1 VVS)	1 (100)	0	-

Other – n (%) (n=1 VVS)	0	1 (100)	1 (100)
Laboratory – n (%)	1 (50)	1 (100)	1 (100)
**Treatment**			
Add-on of non-pharmacological measures – n (%)	0	1 (100)	1 (100)
Add-on of pharmacological measures – n (%)	0	0	0
Increase of pharmacological measures – n (%)	0	0	0
**Follow-up**			
Available – n (%)	1 (50)	0	0
Follow-up-time – months (n=1 VVS)	7	-	-
Improved symptomatic course – n (%) (n=1 VVS)	0	-	-

Quantitative values are shown as mean±SD or median [25th, 75th percentile]; qualitative variables as n (%). Distribution has been assessed with the Shapiro-Wilk test. Abbreviations: CAD, cardiovascular autonomic disorders; COVID-19, coronavirus disease 2019; dOH, delayed orthostatic hypotension; N/n, number; tOH, transient OH; VVS, vasovagal syncope.

STrengthening the **R**eporting of **OB**servational studies in **E**pidemiology (**STROBE**) **Statement** version 4

	Item No	Recommendation	
**Title and abstract**	1	(*a*) Indicate the study's design with a commonly used term in the title or the abstract.	✓
		(*b*) Provide in the abstract an informative and balanced summary of what was done and what was found.	✓
**Introduction**			
Background/rationale	2	Explain the scientific background and rationale for the investigation being reported.	✓
Objectives	3	State specific objectives, including any prespecified hypotheses.	✓/ ND (we did not include a prespecified hypotheses)
**Methods**			
Study design	4	Present key elements of study design early in the paper.	✓
Setting	5	Describe the setting, locations, and relevant dates, including periods of recruitment, exposure, follow-up, and data collection.	✓
Participants	6	Give the eligibility criteria, and the sources and methods of selection of participants. Describe methods of follow-up.	✓
Variables	7	Clearly define all outcomes, exposures, predictors, potential confounders, and effect modifiers. Give diagnostic criteria, if applicable.	✓ (if applicable)
Data sources/ measurement	8	For each variable of interest, give sources of data and details of methods of assessment (measurement).	✓
Bias	9	Describe any efforts to address potential sources of bias.	✓
Study size	10	Explain how the study size was arrived at.	✓
Quantitative variables	11	Explain how quantitative variables were handled in the analyses. If applicable, describe which groupings were chosen and why.	✓
Statistical methods	12	(*a*) Describe all statistical methods, including those used to control for confounding.	✓ (if applicable)
		(*b*) Describe any methods used to examine subgroups and interactions.	✓
		(*c*) Explain how missing data were addressed.	✓
		(*d*) If applicable, explain how loss to follow-up was addressed.	✓
		(*e*) Describe any sensitivity analyses.	ND

**Results**			
Participants	13	(a) Report numbers of individuals at each stage of study—e.g., numbers potentially eligible, examined for eligibility, confirmed eligible, included in the study, completing follow-up, and analysed.	✓
		(b) Give reasons for non-participation at each stage.	✓
		(c) Consider use of a flow diagram.	✓
Descriptive data	14	(a) Give characteristics of study participants (e.g., demographic, clinical, social) and information on exposures & potential confounders.	✓ (if applicable)
		(b) Indicate number of participants with missing data for each variable of interest.	✓
		(c) Summarise follow-up time (e.g., average and total amount).	✓
Outcome data	15	Report numbers of outcome events or summary measures over time.	✓
Main results	16	(*a*) Give unadjusted estimates and, if applicable, confounder-adjusted estimates and their precision (e.g., 95% confidence interval). Make clear which confounders were adjusted for and why they were included.	NA
		(*b*) Report category boundaries when continuous variables were categorized.	✓
		(*c*) If relevant, consider translating estimates of relative risk into absolute risk for a meaningful time period.	NA
Other analyses	17	Report other analyses done—e.g., analyses of subgroups and interactions, and sensitivity analyses.	✓ (if applicable)
**Discussion**			
Key results	18	Summarise key results with reference to study objectives.	✓
Limitations	19	Discuss limitations of the study, taking into account sources of potential bias or imprecision. Discuss both direction and magnitude of any potential bias.	✓
Interpretation	20	Give a cautious overall interpretation of results considering objectives, limitations, multiplicity of analyses, results from similar studies, and other relevant evidence.	✓
Generalisability	21	Discuss the generalisability (external validity) of the study results.	✓
**Other information**			
Funding	22	Give the source of funding and the role of the funders for the present study and, if applicable, for the original study on which the present article is based.	✓

Abbreviations: NA, not applicable; ND, not done.

References

1. Vernino S, Bourne KM, Stiles LE, Grubb BP, Fedorowski A, Stewart JM, et al. Postural orthostatic tachycardia syndrome (POTS): State of the science and clinical care from a 2019 National Institutes of Health Expert Consensus Meeting - Part 1. Auton Neurosci. 2021;235:102828. doi: 10.1016/j.autneu.2021.102828.
2. Freeman R, Wieling W, Axelrod FB, Benditt DG, Benarroch E, Biaggioni I, et al. Consensus statement on the definition of orthostatic hypotension, neurally mediated syncope and the postural tachycardia syndrome. Clin Auton Res. 2011;21(2):69-72. doi: 10.1007/s10286-011-0119-5.
3. Brignole M, Moya A, de Lange FJ, Deharo JC, Elliott PM, Fanciulli A, et al. 2018 ESC Guidelines for the diagnosis and management of syncope. Eur Heart J. 2018;39(21):1883-948. doi: 10.1093/eurheartj/ehy037.
4. van Wijnen VK, Finucane C, Harms MPM, Nolan H, Freeman RL, Westerhof BE, et al. Noninvasive beat-to-beat finger arterial pressure monitoring during orthostasis: a comprehensive review of normal and abnormal responses at different ages. J Intern Med. 2017;282(6):468-83. doi: 10.1111/joim.12636.
5. WHO Guidelines Approved by the Guidelines Review Committee. Clinical management of COVID-19: Living guideline. Geneva: World Health Organization © World Health Organization 2021; 2022.
6. Low DA, da Nóbrega AC, Mathias CJ. Exercise-induced hypotension in autonomic disorders. Auton Neurosci. 2012;171(1-2):66-78. doi: 10.1016/j.autneu.2012.07.008.
7. Jansen RW, Lipsitz LA. Postprandial hypotension: epidemiology, pathophysiology, and clinical management. Ann Intern Med. 1995;122(4):286-95. doi: 10.7326/0003-4819-122-4-199502150-00009.
8. Owens PE, Lyons SP, O'Brien ET. Arterial hypotension: prevalence of low blood pressure in the general population using ambulatory blood pressure monitoring. J Hum Hypertens. 2000;14(4):243-7. doi: 10.1038/sj.jhh.1000973.
9. Parati G, Stergiou G, O'Brien E, Asmar R, Beilin L, Bilo G, et al. European Society of Hypertension practice guidelines for ambulatory blood pressure monitoring. J Hypertens. 2014;32(7):1359-66. doi: 10.1097/hjh.0000000000000221.
10. Rivasi G, Groppelli A, Brignole M, Soranna D, Zambon A, Bilo G, et al. Association between hypotension during 24 h ambulatory blood pressure monitoring and reflex syncope: the SynABPM 1 study. Eur Heart J. 2022;43(38):3765-76. doi: 10.1093/eurheartj/ehac347.
11. Brignole M, Rivasi G, Fedorowski A, Ståhlberg M, Groppelli A, Ungar A. Tests for the identification of reflex syncope mechanism. Expert Rev Med Devices. 2023;20(2):109-19. doi: 10.1080/17434440.2023.2174428.
12. Novak P. Quantitative autonomic testing. J Vis Exp. 2011(53). doi: 10.3791/2502.
